# Role of intratumoral and peritumoral CT radiomics for the prediction of EGFR gene mutation in primary lung cancer

**DOI:** 10.1259/bjr.20220374

**Published:** 2022-09-22

**Authors:** Motohiko Yamazaki, Takuya Yagi, Masaki Tominaga, Kojiro Minato, Hiroyuki Ishikawa

**Affiliations:** ^1^ Department of Radiology and Radiation Oncology, Niigata University Graduate School of Medical and Dental Sciences, Niigata, Japan

## Abstract

**Objectives:**

To determine the added value of combining intratumoral and peritumoral CT radiomics for the prediction of epidermal growth factor receptor (EGFR) gene mutations in primary lung cancer (PLC).

**Methods:**

This study included 478 patients with PLC (348 adenocarcinomas and 130 other histological types) who underwent surgical resection and EGFR gene testing. Two radiologists performed segmentation of tumors and peritumoral regions using precontrast high-resolution CT images, and 398 radiomic features (212 intra- and 186 peritumoral features) were extracted. The peritumoral region was defined as the lung parenchyma within a distance of 3 mm from the tumor border. Model performance was estimated using Random Forest, a machine-learning algorithm.

**Results:**

EGFR mutations were found in 162 tumors; 161 adenocarcinomas, and one pleomorphic carcinoma. After exclusion of poorly reproducible and redundant features, 32 radiomic features remained (14 intra- and 18 peritumoral features) and were included in the model building. For predicting EGFR mutations, combining intra- and peritumoral radiomics significantly improved the performance compared to intratumoral radiomics alone (AUC [area under the receiver operating characteristic curve], 0.774 *vs* 0.730; *p* < 0.001). Even in adenocarcinomas only, adding peritumoral radiomics significantly increased performance (AUC, 0.687 *vs* 0.630; *p* < 0.001). The predictive performance using radiomics and clinical features was significantly higher than that of clinical features alone (AUC, 0.826 *vs* 0.777; *p* = 0.005).

**Conclusions:**

Combining intra- and peritumoral radiomics improves the predictive accuracy of EGFR mutations and could be used to aid in decision-making of whether to perform biopsy for gene tests.

**Advances in knowledge:**

Adding peritumoral to intratumoral radiomics yields greater accuracy than intratumoral radiomics alone in predicting EGFR mutations and may serve as a non-invasive method of predicting of the gene status in PLC.

## Introduction

Epidermal growth factor receptor (EGFR) gene mutations are oncogenic driver mutations in primary lung cancer (PLC) and are frequently observed in non-smoking Asian females.^1,2^ The discovery of EGFR mutations has brought a revolutionary change to PLC treatment because mutated tumors respond well to EGFR tyrosine kinase inhibitors (TKIs).^3^

Tissue examination is required to confirm EGFR mutations; however, biopsy is invasive and carries a risk of complications. As such, CT examination, which is a non-invasive and readily available tool, plays an important role in predicting mutations.

Radiomics is an image analysis methodology that extracts a large number of quantitative features that cannot be assessed by the human eye,^4^ and has recently garnered considerable attention in predicting EGFR mutations.^5–8^ In oncology, radiomics is mainly divided into intra- and peritumoral radiomics. Nevertheless, many previous studies have focused on only intratumoral radiomic features in the prediction of EGFR mutations.^5–7^ Therefore, the importance of peritumoral radiomic features in detecting mutations is not well known. Given that peritumoral radiomics is associated with many aspects of PLC, such as prognosis,^9–12^ histology,^13,14^ and response to chemotherapy,^15,16^ we hypothesized that peritumoral as well as intratumoral features would provide useful information for predicting EGFR mutations.

The purpose of this study was to determine the added value of combining intra- and peritumoral CT radiomics for the prediction of EGFR mutations in PLC.

## Methods and materials

### Patients

This single institution retrospective study was conducted in accordance with the Declaration of Helsinki and was approved by the Institutional Ethics Committee (approved number, 2020–0420). Requirement for informed consent was waived because of the retrospective nature of this study. A total of 599 consecutive patients with PLC who underwent surgical resection between November 2015 and December 2020 were considered for this study. We excluded 121 patients for the following reasons: (1) EGFR gene testing was not performed (*n* = 66); (2) precontrast high-resolution CT (HRCT) was not performed within three months preoperatively at our institution (*n* = 43); (3) received chemotherapy or radiotherapy preoperatively (*n* = 10); and (4) unclear tumor margin on HRCT (*n* = 2). Finally, 478 patients with 478 PLCs (288 men and 190 women; median age, 70 years; range, 29–87 years) were included in the present study. Reasons for detecting lung cancers were various, including health checkup, associated symptoms, and incidental detection. Before surgery, chest and abdominal CT, brain CT or MRI, and 18F-fluorodeoxyglucose PET/CT were carried out to determine the clinical stage.

### CT acquisition

Imaging was performed using one of the following three CT scanners: (1) SOMATOM Force (Siemens, Germany), (2) Aquilion ONE (Canon, Japan), or (3) Ingenuity Elite (Philips, the Netherlands). Of the machines, the SOMATOM Force was mainly used (317/478, 66.3%). After patients were placed in the supine position, scanning from the lung apex to the base was performed during the deep inspiratory phase. Most patients received both pre- and postcontrast CTs for preoperative staging. However, eight patients underwent only precontrast CT because of renal dysfunction or iodine allergy. Although postcontrast CT is the standard for lung cancer staging, we routinely perform both pre- and postcontrast CTs unless there is contraindication for contrast agents because adding precontrast CT allows to accurately evaluate the degree of lesions' enhancement. When a preoperative biopsy cannot confirm the diagnosis of lung nodules, the degree of enhancement can be useful to characterize them.^17^ Adding precontrast CT also has the benefit in differentiating between high-density mediastinal cysts and lymph node enlargement which can be misleading on only postcontrast CT. In this study, precontrast HRCT images were used for the analysis. The scanning parameters for HRCT were as follows: tube current, automatic exposure control; tube voltage, 120 kVp; detector pitch, 0.813–1.172 mm; detector collimation, 64–96 × 0.5–0.625 mm; gantry rotation time, 0.4–0.5 s; field of view, 200 × 200 mm; pixel spacing, 512 × 512; and reconstruction slice thickness, 1.0 mm. A sharp algorithm was used for all the reconstruction factors.

### Gene testing

Gene testing was performed on specimens acquired either by surgery (*n* = 330) or transbronchoscopy (*n* = 148). Various gene testing techniques were used in this retrospective study, but most (403/478, 84.3%) were polymerase chain reaction clamp-based test.^18^

### Radiomic analysis

**Figure 1.**
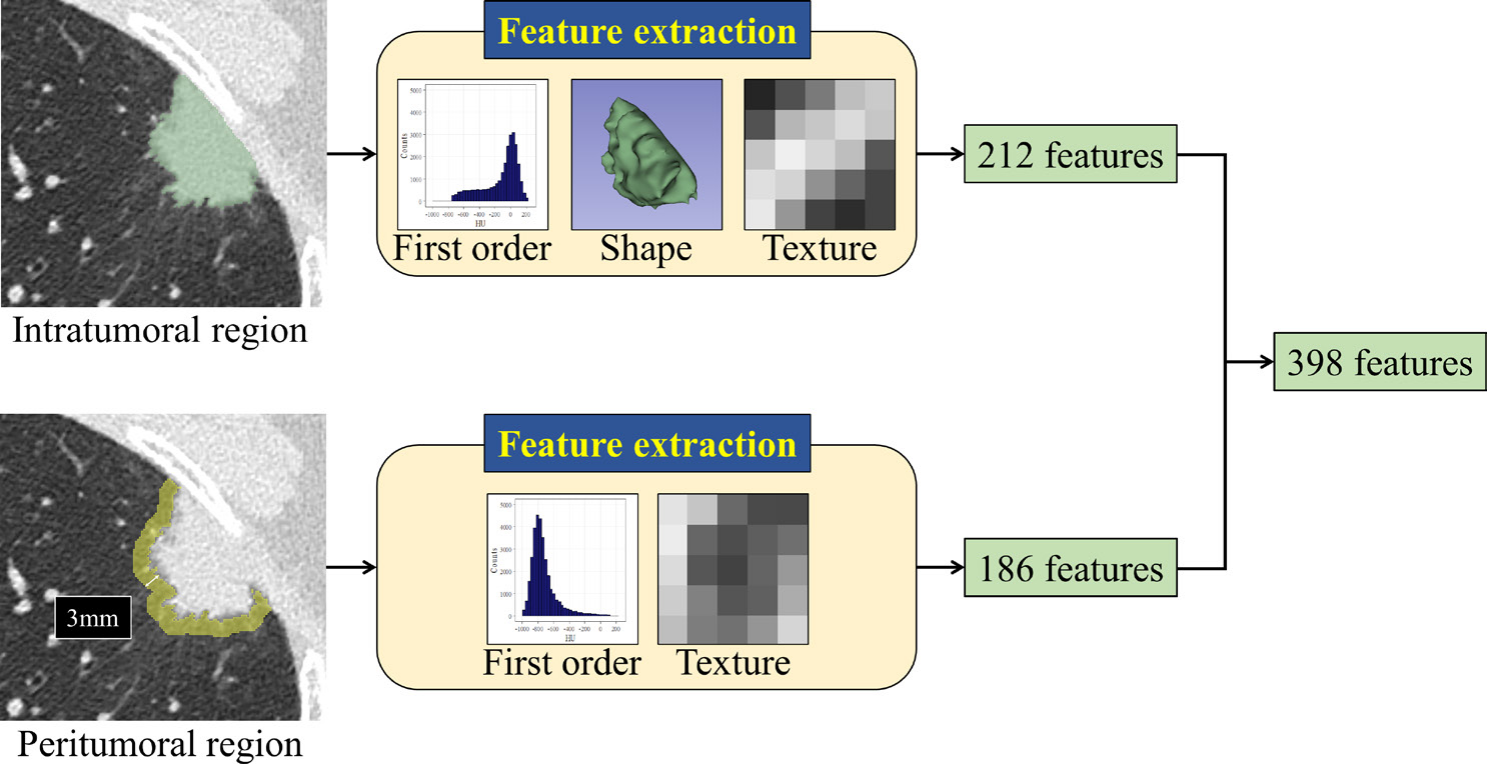
The process of feature extraction. A total of 398 radiomic features with 212 intratumoral (first order statistic, shape, and texture features) and 186 peritumoral features (first order statistic and texture features) were extracted from both the whole tumor (3D analysis) and the maximum cross-section of the tumor (2D analysis). The peritumoral region was defined as the lung parenchyma within 3 mm from the tumor margin.

The acquired CT images were anonymized and then transferred to a personal computer that was not connected to the network. One radiologist with 17 years of experience semi-automatically segmented the tumor and a peritumoral region using Segmentation Editor module^19^ implemented in 3D Slicer^20^ version 4.13.0. The peritumoral region was defined as the lung parenchyma within a distance of 3 mm from the tumor border, which included air in the lung, pulmonary vessels, and bronchi, but not the thoracic wall and mediastinum. This criterion was based on a previous report that the peritumoral lung parenchyma within 3 mm without including the thoracic wall or mediastinum was significantly related to overall survival in lung cancer.^10^ A detailed procedure for segmentation is described in electronic Supplementary Document. After segmentation, a total of 398 radiomic features were computed using SlicerRadiomics,^21^ an extension for 3D Slicer, from the whole tumor (3D analysis) and the maximum cross-section of the tumor (2D analysis). The analyzed radiomic features comprised 212 intra- and 186 peritumoral features, including first order statistic, shape, and texture features. A fixed bin width of 25 Hounsfield units was used for the texture analysis, which is the default value of 3D Slicer. The description of each radiomic feature is available via Pyradiomics Documentation: https://pyradiomics.readthedocs.io/en/latest/features.html. To evaluate the interobserver agreement of radiomic feature values, another radiologist with 15 years of experience segmented 50 tumors randomly selected from the study group. Figure 1 illustrates the feature extraction process.

### Unsupervised clustering analysis

Before feature selection and model building, unsupervised hierarchical clustering analysis was conducted to identify differences in the overall expression pattern of intra- and peritumoral radiomic features depending on the presence or absence of EGFR mutations. Ward’s method based on the SCC was used as the clustering algorithm.^22^ To adjust the feature scale, each radiomic feature was normalized to a mean of 0 and standard deviation of 1. ComplexHeatmap package^23^ implemented in R software^24^ version 4.0.4 was used to draw the heatmap.

### Building the machine-learning model

As some radiomic features are unstable and similar to each other, poorly-reproducible and highly-correlated features were excluded using the criteria of the intraclass correlation coefficient (ICC)<0.5 and Spearman’s correlation coefficient (SCC)≥0.7. ICC calculation was based on the ICC (2, 1).^25^ After feature selection, the following four Random Forest classifiers^26^ were built for predicting EGFR mutations: (1) a model with intratumoral radiomics alone, (2) a model combining intra- and peritumoral radiomics, (3) a model combining intra- and peritumoral radiomics and clinical features, and (4) a model with clinical features alone. The ranger R package^27^ implemented in R software was used for model building. Clinical features included age, sex, Brinkman index, and clinical stage. The Brinkman index was calculated as the number of cigarettes smoked per day multiplied by the number of years smoked.^28^

Random Forest is an ensemble machine-learning algorithm consisting of many decision trees^26^ ; decision tree is supervised learning technique with tree-like structure. Random Forest integrates all predictions made by decision trees into one prediction. In this study, 500 decision trees were generated in Random Forest. For each tree, training data with the same number of the entire dataset (*n* = 478) were obtained by sampling with replacement from the entire dataset. Because of the resampling, about one-third of the patients (n ≈ 159) were not selected for each sampling. The unselected dataset is called out-of-bag (OOB) data and was used as test data to estimate the prediction performance for unseen data in this study. Predictions based on OOB data are reportedly an unbiased estimator of the performance on future data.^26^ The number of features to be randomly selected when splitting the patient group was set to the square root of the total number of features; this is the default setting of the ranger R package. The other hyperparameters were also set to the default value. After constructing 500 trees, each tree’s prediction was combined, and the predicted probability of EGFR mutations was determined for each patient. Furthermore, we measured feature importance according to the mean decrease in the Gini index.^29^ A decrease in the Gini index indicates how a feature contributes to discrimination between positive and negative EGFR mutations. The larger the mean decrease in the Gini index, the greater the feature contributes to the discrimination.

### Statistical analysis

Fisher’s exact test and Mann–Whitney U-test were used to assess statistical differences in categorical and continuous variables, respectively. The performance of the predictive model using OOB data was evaluated using the area under the receiver operating characteristic curve (AUC). We investigated whether adding peritumoral radiomics significantly improved the AUC using the DeLong method.^30^ Namely, a comparison of the AUCs was a one-sided hypothesis test, in which the null hypothesis was an assertion that a model with peritumoral radiomics was not superior to a model without. The other statistical analyses were two-tailed. A calibration curve was delineated to assess the agreement between the predicted and the actual probabilities of EGFR mutations on OOB data. The goodness-of-fit of the model was determined using the Hosmer-Lemeshow test. Statistical significance was set at *p* < 0.05.

## Results

### Characteristics of patients and tumors

EGFR mutations were detected in 162 (33.9%) patients, and mutations were negative in 316 (66.1%) patients. As for race, one patient was a Caucasian, and the others were East Asian. Table 1 describes the characteristics of the patients and tumors based on their EGFR status. The percentage of EGFR mutations was significantly higher in younger patients (*p* = 0.010), female sex (*p* < 0.001), lower Brinkman Index (*p* < 0.001), and early clinical stage (*p* < 0.001). All mutated tumors were adenocarcinomas except for one case of pleomorphic carcinoma. Table 2 summarizes the histological types of adenocarcinomas. EGFR-mutated adenocarcinomas were lepidic predominant, papillary, or acinar types (*p* < 0.001); no other histological types showed mutations.

**Table 1.** Patient and tumor characteristics (*n* = 478)

Variables	EGFR-mutant (*n* = 162)	Wild-type (*n* = 316)	*P*
Age			0.010
< 65 y	52 (32.1)	67 (21.2)	
≥ 65 y	110 (67.9)	249 (78.8)	
Sex			< 0.001
Males	51 (31.5)	237 (75.0)	
Females	111 (68.5)	79 (25.0)	
Brinkman index			< 0.001
< 400	122 (75.3)	85 (26.9)	
≥ 400	40 (24.7)	231 (73.1)	
Clinical stage			< 0.001
0 or I	154 (95.1)	246 (77.8)	
II or III	8 (4.9)	70 (22.2)	
Histologic type			
Adenocarcinoma (*n* = 348)	161 (99.4)	187 (59.2)	
Squamous cell carcinoma (*n* = 96)	0 (0)	96 (30.4)	
Other types (*n* = 34)^a^	1 (0.6)	33 (10.4)	

EGFR, epidermal growth factor receptor.

Data are presented as numbers (%).

a Eleven neuroendocrine carcinomas, eight adenosquamous cell carcinomas, five pleomorphic carcinomas, three large cell carcinomas, three carcinosarcomas, three carcinoids, and one mucoepidermoid carcinoma.

**Table 2.** Adenocarcinoma types (*n* = 348)

Histologic type	EGFR-mutant (*n* = 161)	Wild-type (*n* = 187)	*P*
Lepidic predominant adenocarcinoma^a^	51 (31.7)	45 (24.1)	
Papillary or acinar adenocarcinoma	110 (68.3)	92 (49.2)	
Other adenocarcinomas^b^	0 (0)	50 (26.7)	< 0.001

EGFR, epidermal growth factor receptor.

Data are presented as numbers (%).

a Adenocarcinoma *in situ*, minimally invasive adenocarcinoma, or lepidic adenocarcinoma

b Solid, invasive mucinous, enteric, micropapillary, or colloid adenocarcinoma

### Clustering analysis

**Figure 2.**
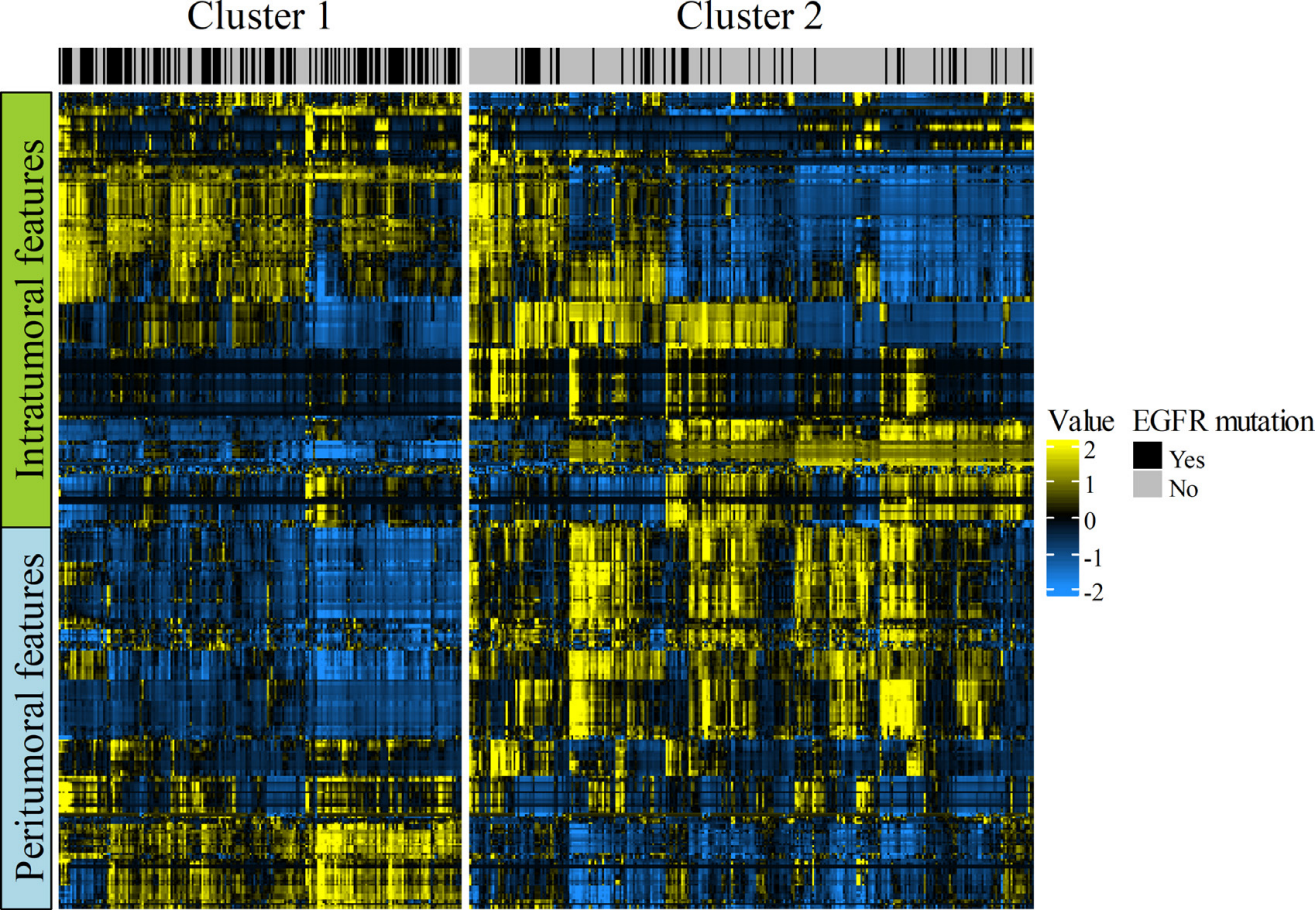
Radiomic heatmap with 478 patients on the x-axis and 398 radiomic features (212 intra- and 186 peritumoral features) on the y-axis. The unsupervised clustering analysis identified two patient clusters showing similar radiomic expression patterns. These clusters showed significant different EGFR mutation rates [56.3% (112/199) for cluster 1 *vs* 17.9% (50/279) for cluster 2; *p* < 0.001]; this result represents the association between radiomics and EGFR mutations. Note that both the intra- and peritumoral features visually show different heatmaps between the two clusters.

Figure 2 illustrates the radiomic heatmap constructed by using unsupervised clustering analysis. The algorithm divided the 478 patients into two clusters with similar radiomic features. These two clusters were found to have significantly different EGFR mutation rates (cluster 1, 56.3% [112/199] *vs* cluster 2, 17.9% [50/279]; *p* < 0.001), indicating that intra- and peritumoral radiomic features were significantly associated with EGFR mutations.

### Radiomic feature selection

**Figure 3.**
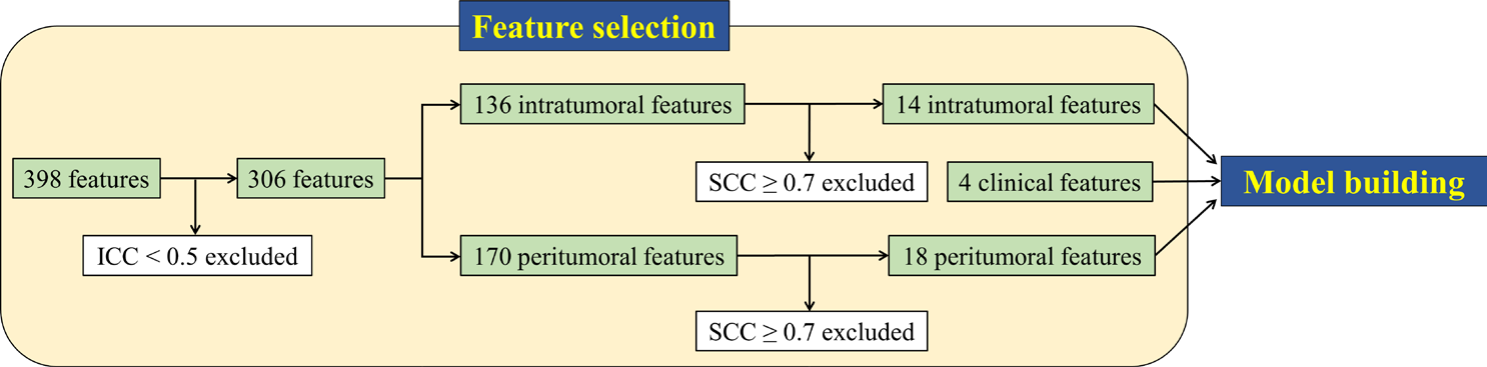
Feature selection algorithm using intraclass correlation coefficient (ICC) and Spearman’s correlation coefficient (SCC). A total of 32 features (14 intra- and 18 peritumoral features) remained after excluding low reproducible (ICC<0.5) and redundant features (SCC≥0.7). The 32 radiomic and four clinical features (age, sex, Brinkman index, and clinical stage) were subsequently included in the predictive model.

Figure 3 shows a flow chart of the feature selection. Of the 398 radiomic features, 92 were excluded because of their low reproducibility (ICC<0.5). Among the remaining 306 features, 274 were subsequently removed due to redundancy (SCC≥0.7). Consequently, 32 radiomic features with 14 intra- and 18 peritumoral features remained and were used for model building. The selected 32 features consisted of three shape features, nine first order statistics, and 20 texture features, and they are presented in Table 3. All 398 radiomic features and ICCs are listed in Supplementary Table 1.

**Table 3.** The last remaining radiomic features (*n* = 32)

Feature name	Dimension	Feature family	Extracted region
Elongation	2D	Shape	Intratumor
Maximum	2D	First order	Intratumor
Cluster shade	2D	GLCM	Intratumor
Large area low gray level emphasis	2D	GLSZM	Intratumor
Elongation	3D	Shape	Intratumor
Sphericity	3D	Shape	Intratumor
90th percentile	3D	First order	Intratumor
Energy	3D	First order	Intratumor
Maximum	3D	First order	Intratumor
Maximal correlation coefficient	3D	GLCM	Intratumor
Gray level non-uniformity	3D	GLSZM	Intratumor
Small area emphasis	3D	GLSZM	Intratumor
Zone variance	3D	GLSZM	Intratumor
Coarseness	3D	NGTDM	Intratumor
10th percentile	2D	First order	Peritumor
Maximum	2D	First order	Peritumor
Minimum	2D	First order	Peritumor
Cluster shade	2D	GLCM	Peritumor
Difference variance	2D	GLCM	Peritumor
Inverse difference moment normalized	2D	GLCM	Peritumor
Maximal correlation coefficient	2D	GLCM	Peritumor
Large area low gray level emphasis	2D	GLSZM	Peritumor
Coarseness	2D	NGTDM	Peritumor
Strength	2D	NGTDM	Peritumor
Energy	3D	First order	Peritumor
Maximum	3D	First order	Peritumor
Cluster shade	3D	GLCM	Peritumor
Inverse difference moment normalized	3D	GLCM	Peritumor
Maximal correlation coefficient	3D	GLCM	Peritumor
Large dependence high gray level emphasis	3D	GLDM	Peritumor
Large area high gray level emphasis	3D	GLSZM	Peritumor
Coarseness	3D	NGTDM	Peritumor

GLCM, gray level co-occurrence matrix; GLDM, gray level dependence matrix; GLSZM, gray level size zone matrix; NGTDM, neighboring gray tone difference matrix.

### Model performance for EGFR mutation using OOB data

The model performance for predicting EGFR mutations on OOB data is presented in Table 4. Regarding the prediction among all cases (*n* = 478), intratumoral radiomics showed an AUC of 0.730 (95% confidence interval [CI]: 0.682–0.777). Compared to this performance, combined intra- and peritumoral radiomics showed a significantly higher performance, with an AUC of 0.774 (95% CI: 0.730–0.817; *p* < 0.001). The AUC for adding clinical features to radiomics was 0.826 (95% CI: 0.788–0.864), which was significantly higher than the AUC of 0.777 (95% CI: 0.734–0.821) for clinical features alone (*p* = 0.005).

**Table 4.** Model performance in EGFR mutation prediction using OOB data

	AUC (95% CI)^a^	P for difference in AUC
All cases (*n* = 478)		
Intratumor	0.730 (0.682–0.777)	
Intra+peritumor	0.774 (0730–0.817)	< 0.001 ( *vs* intratumor)
Intra+peritumor+clinical	0.826 (0.788–0.864)	0.005 ( *vs* clinical)
Clinical	0.777 (0.734–0.821)	
Adenocarcinoma only (*n* = 348)		
Intratumor	0.630 (0.572–0.689)	
Intra+peritumor	0.687 (0.632–0.743)	< 0.001 ( *vs* intratumor)
Intra+peritumor+clinical	0.744 (0.693–0.796)	0.045 ( *vs* clinical)
Clinical	0.703 (0.648–0.758)	

AUC, area under the curve; CI, confidence interval; EGFR, epidermal growth factor receptor; OOB, out-of-bag.

a AUC was calculated from OOB data, namely a cohort that was not used in building the model.

In terms of performance for adenocarcinoma cases only (*n* = 348), the results were similar. The AUC of intratumoral radiomics was 0.630 (95% CI: 0.572–0.689), and the combination of intra- and peritumoral radiomics showed a significantly increased AUC of 0.687 (95% CI, 0.632–0.743; *p* < 0.001). The AUC by including radiomics and clinical features was 0.744 (95% CI, 0.693–0.796). This performance was significantly higher than the AUC of 0.703 (95% CI: 0.648–0.758) for the clinical features alone (*p* = 0.045).

**Figure 4.**
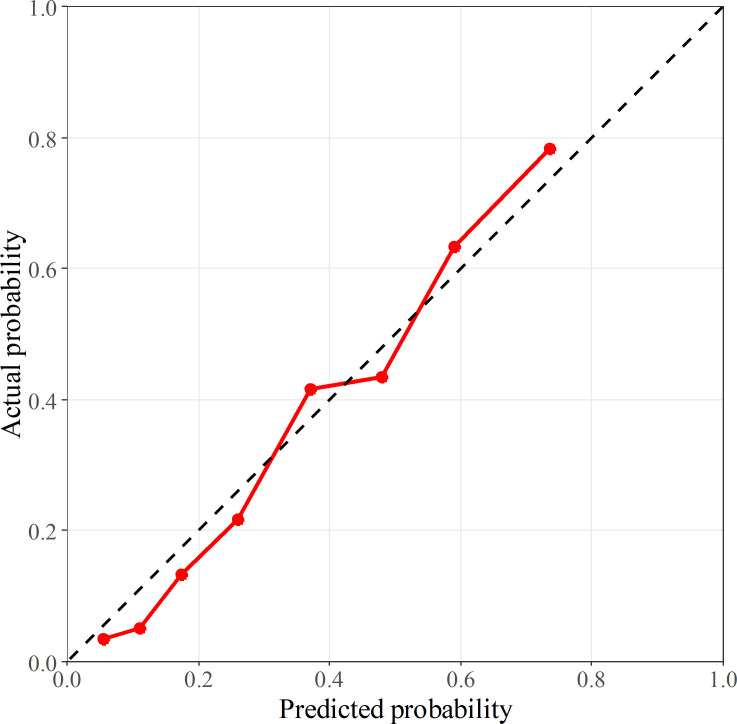
Calibration curve of the predictive model consisted of intratumoral, peritumoral, and clinical features. The x- and y-axes correspond to the probability of EGFR mutations predicted by the model and the actual probability, respectively; the probabilities are based on the out-of-bag data. The model fit well according to the Hosmer-Lemeshow test (*p* = 0.456). The black-dashed line represents perfect calibration.

The calibration curve using OOB data is shown in Figure 4. The Hosmer-Lemeshow test indicated that the model incorporating radiomics and clinical features showed proper calibration (*p* = 0.456).

### Model performance on identical CT scanner

As this study used three different CT scanners, the model performance within patients who were investigated using SOMATOM Force only (*n* = 317) was evaluated to determine the generality of the study results. The AUC (95% CI) using intra- and peritumoral radiomics was significantly higher than that using intratumoral radiomics alone (0.780 [0.727–0.832] *vs* 0.740 [0.684–0.795]; *p* = 0.003), showing similar results.

### Feature importance

**Figure 5.**
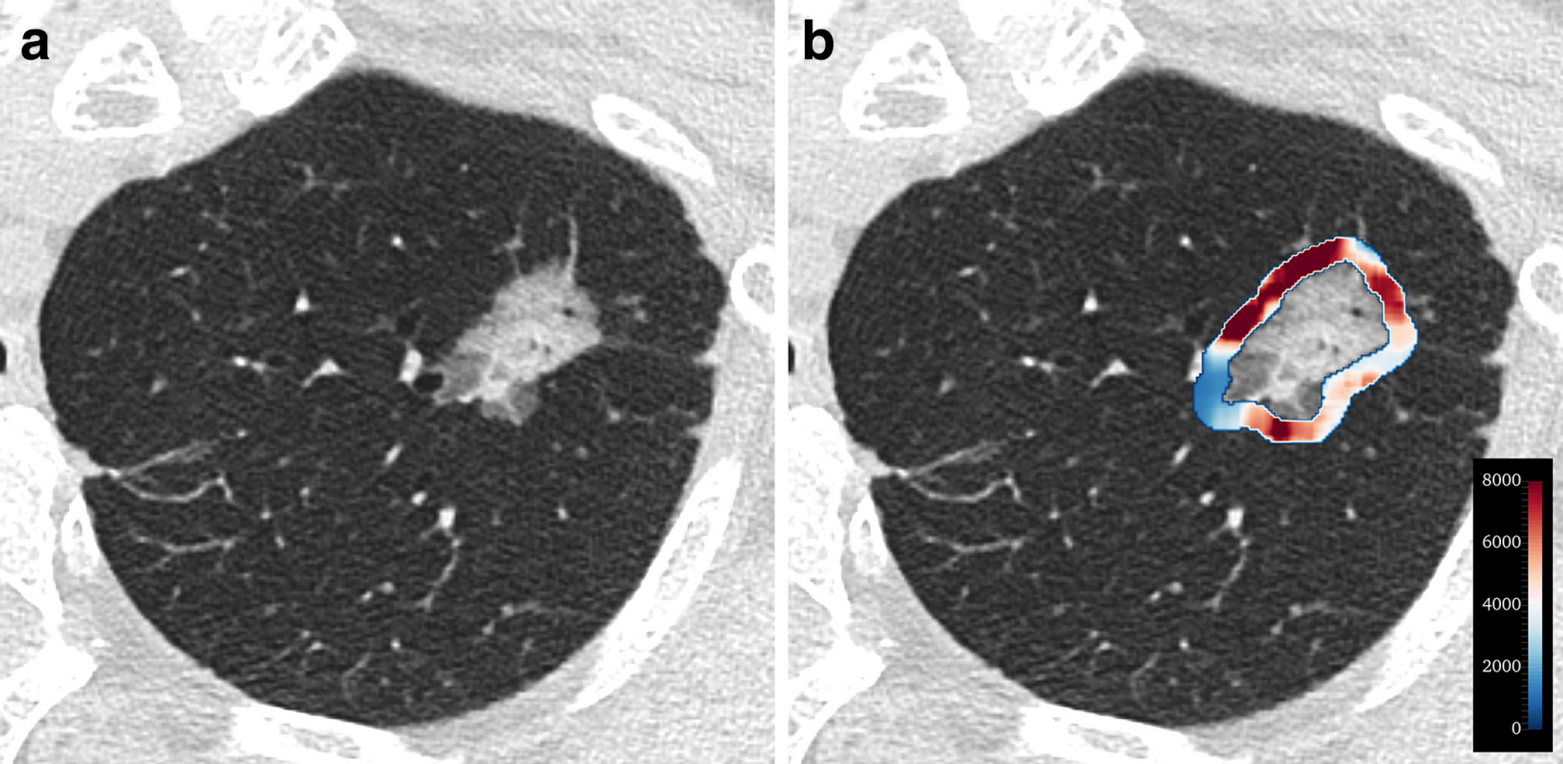
A 72-year-old female (non-smoker) with EGFR-mutant acinar adenocarcinoma. (**a**)Axial CT image shows a part-solid nodule in the apex of the left lung. (**b**)Heatmap that captures a peritumoral feature (large area high gray level emphasis) indicates high expression. The probability of an EGFR mutation predicted by the model was as high as 88.4%.

**Figure 6.**
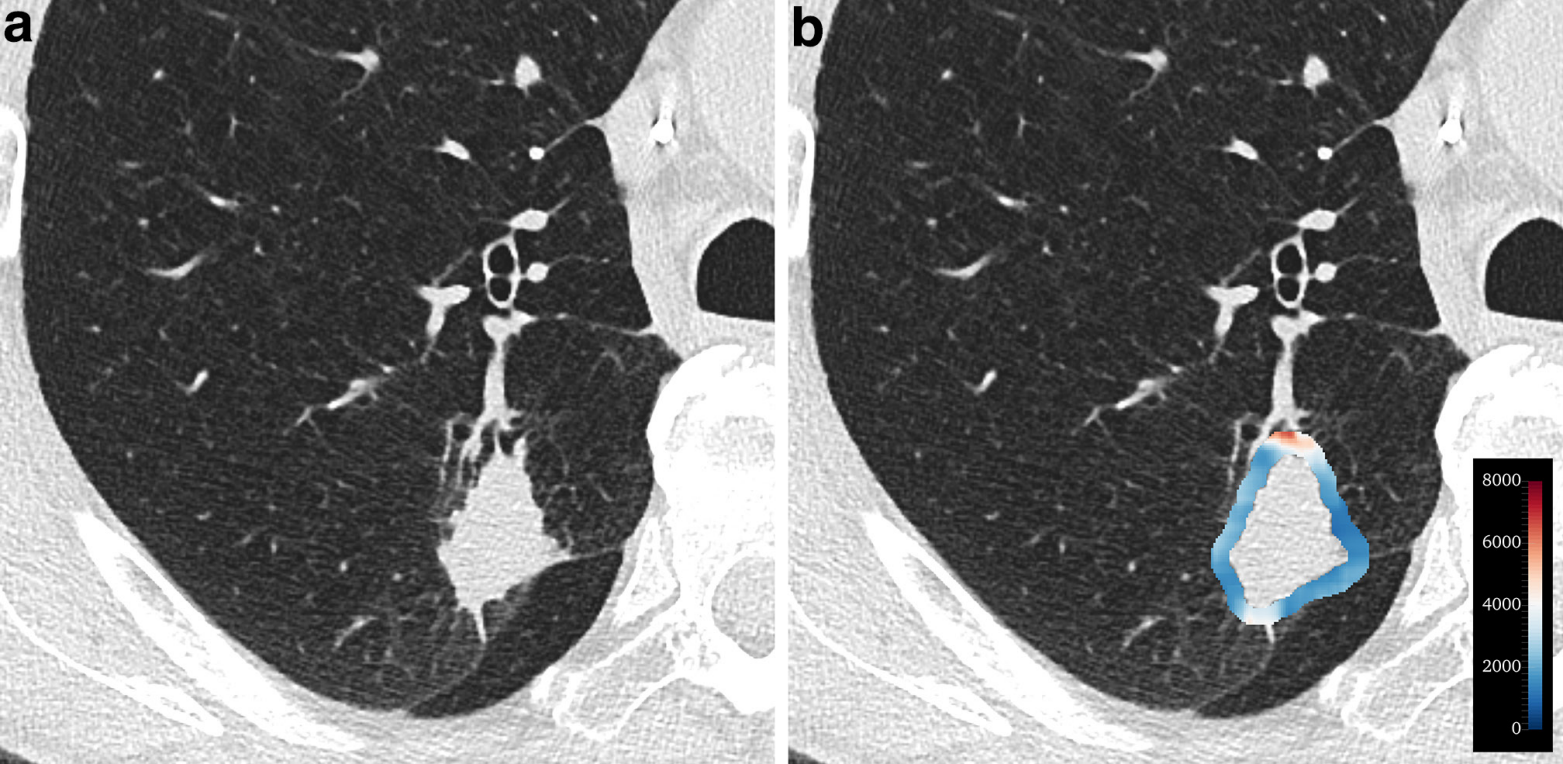
A 74-year-old male (smoker) with EGFR-wild-type solid adenocarcinoma. (**a**)Axial CT image shows a solid nodule in the right upper lobe. Emphysema is present around the tumor. (**b**)Heatmap that captures a peritumoral feature (large area high gray level emphasis) indicates low expression. The probability of an EGFR mutation predicted by the model was as low as 2.1%.

The top 10 most important features were eight radiomic features, Brinkman index, and sex (Table 5). Brinkman index and sex were ranked as the first and third most important features, respectively. Among the radiomic features, the intratumoral 90th percentile was the most important, followed by peritumoral large area high gray level emphasis (LAHGLE); both were 3D features. Specifically, a low 90th percentile and high LAHGLE were significantly associated with EGFR mutations (*p* < 0.001 for each). The importance of other features is presented in Supplementary Table 2. Figures 5 and 6 show representative cases with and without EGFR mutations.

**Table 5.** Top 10 most important features

Rank	Feature name	Dimension	Feature family	Extracted region	Importance^a^
1	Brinkman index	N.A.	Clinical	N.A.	19.57
2	90th percentile	3D	First order	Intratumor	13.59
3	Sex	N.A.	Clinical	N.A.	11.49
4	LAHGLE	3D	GLSZM	Peritumor	10.25
5	Maximal correlation coefficient	3D	GLCM	Intratumor	6.53
6	Inverse difference moment normalized	2D	GLCM	Peritumor	6.46
7	Cluster shade	2D	GLCM	Intratumor	6.21
8	Inverse difference moment normalized	3D	GLCM	Peritumor	5.52
9	Difference variance	2D	GLCM	Peritumor	5.47
10	Coarseness	3D	NGTDM	Intratumor	4.91

GLCM, gray level co-occurrence matrix; GLSZM, gray level size zone matrix; LAHGLE, large area high gray level emphasis; NGTDM, neighboring gray tone difference matrix.

a Measured using the mean decrease in the Gini index.

## Discussion

The present study constructed a prediction model for EGFR mutations in PLC and found that a combination of intra- and peritumoral radiomics improved accuracy compared to intratumoral radiomics alone. It has also been demonstrated that combining radiomics and clinical features yields greater accuracy than either in isolation in the prediction of EGFR mutations. To our knowledge, few studies on oncology, including but not limited to PLC, have revealed the added value of peritumoral radiomics in predicting cancer gene mutations.

Although biopsy is the gold standard for the diagnosis of gene mutations, understanding the pretest probability using radiomics is clinically useful. For example, if the pretest probability is high, sampling of many tissues can be considered at the time of biopsy to reduce the false-negative rate. Conversely, in cases where the pretest probability is very low, physicians could determine not to perform biopsy intended for gene tests, considering disadvantages such as the risk of complications and increase in medical costs.

All patients in this study had resectable PLC. Although the clinical significance of EGFR mutations has not been established in preoperative patients, gene examinations for such patients may be required for planning systemic drug therapy in case of postoperative recurrence. We believe that radiomic features extracted from primary tumors can be a useful predictor of EGFR mutations in recurrent lesions. Furthermore, a recent systematic review has reported that neoadjuvant EGFR-TKI therapy may be feasible for resectable PLC and that further clinical trials are ongoing.^31^ If strong evidence supports neoadjuvant EGFR-TKI therapy for resectable PLC, our model for predicting EGFR mutation would have a more extensive clinical applicability.

The association between peritumoral radiomics and EGFR mutations in our study is a reasonable result because peritumoral radiomics encompasses various aspects of PLC.^9–16^ For instance, Wu et al^14^ showed that peritumoral radiomics accurately differentiated adenocarcinoma *in situ* (AIS) or minimally invasive adenocarcinoma (MIA) from invasive adenocarcinoma. Regarding the relationship between histology and EGFR status in lung adenocarcinoma, EGFR mutations are mainly observed in AIS, MIA, lepidic, and papillary types; solid and invasive mucinous types rarely show EGFR mutations.^32^ This is in line with our findings, where EGFR mutations were found in either lepidic predominant, papillary, or acinar types.

Only a few studies have used peritumoral radiomics to predict EGFR mutations in PLCs. Choe et al^8^ developed a model using both intra- and peritumoral radiomics to predict EGFR mutations in lung adenocarcinoma. However, the accuracy was not significantly different from that of the model consisting of intratumoral radiomics alone. The discrepancy between the previous results and our own may be explained by differences in the population included in the predictive model. The previous study^8^ trained the predictive model using only lung adenocarcinoma cases, whereas the present study trained by including various histological types. Consequently, our model may have more effectively learned the differences in radiomic features between EGFR-mutated and wild-type tumors.

In our study, the most important peritumoral feature for predicting EGFR mutations was LAHGLE, with high values correlating with mutations. This radiomic feature measures the extent of a large area with a high CT value and can be elevated when high-density structures, such as large pulmonary vessels, are present around tumors. In contrast, LAHGLE can decrease if low-density structures, such as honeycombing and emphysema, exist. According to previous studies, EGFR-mutated adenocarcinomas were more likely to show vascular convergence but less frequently accompanied fibrosis (related to the presence of honeycombing) and emphysema around tumors compared to wild-type adenocarcinomas.^33,34^ These CT features might have affected the LAHGLE values in our study. From a biological perspective, peritumoral radiomics on breast cancer is reported to be associated with the density of tumor-infiltrating lymphocytes, an immune response marker against tumors.^35^ It should be further investigated whether such association is also observed in PLC.

In our study, the intratumoral 90th percentile was the most important radiomic feature in predicting EGFR mutations; a low value was associated with EGFR mutations. A lower 90th percentile value denotes a lower density; therefore, EGFR-mutated tumors in our study may contain CT features with a low density, such as ground-glass opacity and air. This assumption is supported by previous findings,^33,34^ where EGFR-mutated adenocarcinomas had a higher frequency of ground-glass opacity and air-bronchogram than wild-type tumors. The intratumoral 90th percentile is also reported to be useful in the previous study by Yagi et al,^36^ who assessed the histological types of lung adenocarcinoma using texture analysis.

Our study has several limitations. First, it was retrospective and included only surgically resected cases. In addition, all patients except one were East Asian. These can contribute to selection bias. Therefore, the prediction model constructed in this study should be validated for inoperable PLCs and different race groups to clarify the robustness of our model. Second, the present study did not determine whether a distance within 3 mm from the tumor border is optimal for evaluating peritumoral features. Although the previous study also used 3 mm,^10^ ideally, peritumoral radiomic features should be extracted from a variety of ranges to compare their predictive performance. A performance comparison between peritumoral radiomics with and without the chest wall or mediastinum can also be considered. Third, the present study did not assess the performance using postcontrast HRCT. Use of both pre- and postcontrast HRCTs might increase predictive performance, and it should be investigated in future studies.

In conclusion, the combined use of intra- and peritumoral radiomic features improved the performance for EGFR mutation prediction in PLCs. This result may help to consider the indication of EGFR gene tests and to predict candidates for EGFR TKIs.

Supplementary Document.

Supplementary Table 1.

Supplementary Table 2.
