# Supplementary material for: Role of intratumoral and peritumoral CT radiomics for the prediction of EGFR gene mutation in primary lung cancer
Source: Br J Radiol. 2022 Sep 22;95(1140):20220374. doi: 10.1259/bjr.20220374 (PMC9733609; doi:10.1259/bjr.20220374)
Supplement: Supplementary Document. [file bjr.20220374.suppl-01.docx]

***Table S1: All extracted radiomic features (n = 398)***

Note: The finally selected 32 features were denoted with red font.

GLCM, gray level co-occurrence matrix; GLDM, gray level dependence matrix; GLRLM, gray level run length matrix; GLSZM, gray level size zone matrix; ICC, intra-class correlation coefficient; NGTDM, neighboring gray tone difference matrix

| **Feature name** | **Dimension** | **Feature family** | **Extracted region** | **ICC (2, 1)** |
| --- | --- | --- | --- | --- |
| **Elongation** | 2D | Shape | Intratumor | 0.86 |
| Major axis length | 2D | Shape | Intratumor | 0.91 |
| Maximum 2D diameter (column) | 2D | Shape | Intratumor | 0.88 |
| Maximum 2D diameter (row) | 2D | Shape | Intratumor | 0.88 |
| Maximum 2D diameter (slice) | 2D | Shape | Intratumor | 0.86 |
| Maximum 3D diameter | 2D | Shape | Intratumor | 0.86 |
| Mesh volume | 2D | Shape | Intratumor | 0.93 |
| Minor axis length | 2D | Shape | Intratumor | 0.93 |
| Sphericity | 2D | Shape | Intratumor | 0.82 |
| Surface area | 2D | Shape | Intratumor | 0.92 |
| Surface volume ratio | 2D | Shape | Intratumor | 0.64 |
| Voxel volume | 2D | Shape | Intratumor | 0.93 |
| 10th percentile | 2D | First order | Intratumor | 0.73 |
| 90th percentile | 2D | First order | Intratumor | 0.94 |
| Energy | 2D | First order | Intratumor | 0.80 |
| Entropy | 2D | First order | Intratumor | 0.70 |
| Interquartile range | 2D | First order | Intratumor | 0.49 |
| Kurtosis | 2D | First order | Intratumor | 0.28 |
| **Maximum** | 2D | First order | Intratumor | 0.84 |
| Mean absolute deviation | 2D | First order | Intratumor | 0.48 |
| Mean | 2D | First order | Intratumor | 0.89 |
| Median | 2D | First order | Intratumor | 0.92 |
| Minimum | 2D | First order | Intratumor | 0.42 |
| Range | 2D | First order | Intratumor | 0.40 |
| Robust mean absolute deviation | 2D | First order | Intratumor | 0.47 |
| Root mean squared | 2D | First order | Intratumor | 0.81 |
| Skewness | 2D | First order | Intratumor | 0.66 |
| Total energy | 2D | First order | Intratumor | 0.80 |
| Uniformity | 2D | First order | Intratumor | 0.74 |
| Variance | 2D | First order | Intratumor | 0.39 |
| Autocorrelation | 2D | GLCM | Intratumor | 0.14 |
| Cluster prominence | 2D | GLCM | Intratumor | 0.34 |
| **Cluster shade** | 2D | GLCM | Intratumor | 0.59 |
| Cluster tendency | 2D | GLCM | Intratumor | 0.42 |
| Contrast | 2D | GLCM | Intratumor | 0.74 |
| Correlation | 2D | GLCM | Intratumor | 0.69 |
| Difference average | 2D | GLCM | Intratumor | 0.85 |
| Difference entropy | 2D | GLCM | Intratumor | 0.78 |
| Difference variance | 2D | GLCM | Intratumor | 0.54 |
| Inverse difference | 2D | GLCM | Intratumor | 0.91 |
| Inverse difference moment | 2D | GLCM | Intratumor | 0.91 |
| Inverse difference moment normalized | 2D | GLCM | Intratumor | 0.44 |
| Inverse difference normalized | 2D | GLCM | Intratumor | 0.44 |
| Informational measure of correlation 1 | 2D | GLCM | Intratumor | 0.70 |
| Informational measure of correlation 2 | 2D | GLCM | Intratumor | 0.78 |
| Inverse variance | 2D | GLCM | Intratumor | 0.89 |
| Joint average | 2D | GLCM | Intratumor | 0.19 |
| Joint energy | 2D | GLCM | Intratumor | 0.65 |
| Joint entropy | 2D | GLCM | Intratumor | 0.73 |
| Maximal correlation coefficient | 2D | GLCM | Intratumor | 0.56 |
| Maximum probability | 2D | GLCM | Intratumor | 0.71 |
| Sum average | 2D | GLCM | Intratumor | 0.19 |
| Sum entropy | 2D | GLCM | Intratumor | 0.74 |
| Sum of squares | 2D | GLCM | Intratumor | 0.42 |
| Dependence entropy | 2D | GLDM | Intratumor | 0.70 |
| Dependence non-uniformity | 2D | GLDM | Intratumor | 0.94 |
| Dependence non-uniformity normalized | 2D | GLDM | Intratumor | 0.86 |
| Dependence variance | 2D | GLDM | Intratumor | 0.84 |
| Gray level non-uniformity | 2D | GLDM | Intratumor | 0.98 |
| Gray level variance | 2D | GLDM | Intratumor | 0.39 |
| High gray level emphasis | 2D | GLDM | Intratumor | 0.14 |
| Large dependence emphasis | 2D | GLDM | Intratumor | 0.86 |
| Large dependence high gray level emphasis | 2D | GLDM | Intratumor | 0.20 |
| Large dependence low gray level emphasis | 2D | GLDM | Intratumor | 0.57 |
| Low gray level emphasis | 2D | GLDM | Intratumor | 0.30 |
| Small dependence emphasis | 2D | GLDM | Intratumor | 0.84 |
| Small dependence high gray level emphasis | 2D | GLDM | Intratumor | 0.19 |
| Small dependence low gray level emphasis | 2D | GLDM | Intratumor | 0.11 |
| Gray level non-uniformity | 2D | GLRLM | Intratumor | 0.98 |
| Gray level non-uniformity normalized | 2D | GLRLM | Intratumor | 0.73 |
| Gray level variance | 2D | GLRLM | Intratumor | 0.40 |
| High gray level run emphasis | 2D | GLRLM | Intratumor | 0.14 |
| Long run emphasis | 2D | GLRLM | Intratumor | 0.85 |
| Long run high gray level emphasis | 2D | GLRLM | Intratumor | 0.14 |
| Long run low gray level emphasis | 2D | GLRLM | Intratumor | 0.42 |
| Low gray level run emphasis | 2D | GLRLM | Intratumor | 0.24 |
| Run entropy | 2D | GLRLM | Intratumor | 0.68 |
| Run length non-uniformity | 2D | GLRLM | Intratumor | 0.93 |
| Run length non-uniformity normalized | 2D | GLRLM | Intratumor | 0.85 |
| Run percentage | 2D | GLRLM | Intratumor | 0.86 |
| Run variance | 2D | GLRLM | Intratumor | 0.84 |
| Short run emphasis | 2D | GLRLM | Intratumor | 0.85 |
| Short run high gray level emphasis | 2D | GLRLM | Intratumor | 0.14 |
| Short run low gray level emphasis | 2D | GLRLM | Intratumor | 0.19 |
| Gray level non-uniformity | 2D | GLSZM | Intratumor | 0.99 |
| Gray level non-uniformity normalized | 2D | GLSZM | Intratumor | 0.69 |
| Gray level variance | 2D | GLSZM | Intratumor | 0.39 |
| High gray level zone emphasis | 2D | GLSZM | Intratumor | 0.14 |
| Large area emphasis | 2D | GLSZM | Intratumor | 0.83 |
| Large area high gray level emphasis | 2D | GLSZM | Intratumor | 0.22 |
| **Large area low gray level emphasis** | 2D | GLSZM | Intratumor | 0.73 |
| Low gray level zone emphasis | 2D | GLSZM | Intratumor | 0.10 |
| Size zone non-uniformity | 2D | GLSZM | Intratumor | 0.94 |
| Size zone non-uniformity normalized | 2D | GLSZM | Intratumor | 0.81 |
| Small area emphasis | 2D | GLSZM | Intratumor | 0.80 |
| Small area high gray level emphasis | 2D | GLSZM | Intratumor | 0.16 |
| Small area low gray level emphasis | 2D | GLSZM | Intratumor | 0.10 |
| Zone entropy | 2D | GLSZM | Intratumor | 0.65 |
| Zone percentage | 2D | GLSZM | Intratumor | 0.85 |
| Zone variance | 2D | GLSZM | Intratumor | 0.81 |
| Busyness | 2D | NGTDM | Intratumor | 0.53 |
| Coarseness | 2D | NGTDM | Intratumor | 0.48 |
| Complexity | 2D | NGTDM | Intratumor | 0.53 |
| Contrast | 2D | NGTDM | Intratumor | 0.36 |
| Strength | 2D | NGTDM | Intratumor | 0.43 |
| **Elongation** | 3D | Shape | Intratumor | 0.87 |
| Flatness | 3D | Shape | Intratumor | 0.90 |
| Least axis length | 3D | Shape | Intratumor | 0.91 |
| Major axis length | 3D | Shape | Intratumor | 0.89 |
| Maximum 2D diameter (column) | 3D | Shape | Intratumor | 0.90 |
| Maximum 2D diameter (row) | 3D | Shape | Intratumor | 0.80 |
| Maximum 2D diameter (slice) | 3D | Shape | Intratumor | 0.91 |
| Maximum 3D diameter | 3D | Shape | Intratumor | 0.86 |
| Mesh volume | 3D | Shape | Intratumor | 0.93 |
| Minor axis length | 3D | Shape | Intratumor | 0.94 |
| **Sphericity** | 3D | Shape | Intratumor | 0.55 |
| Surface area | 3D | Shape | Intratumor | 0.82 |
| Surface volume ratio | 3D | Shape | Intratumor | 0.89 |
| Voxel volume | 3D | Shape | Intratumor | 0.93 |
| 10th percentile | 3D | First order | Intratumor | 0.69 |
| **90th percentile** | 3D | First order | Intratumor | 0.98 |
| **Energy** | 3D | First order | Intratumor | 0.73 |
| Entropy | 3D | First order | Intratumor | 0.68 |
| Interquartile range | 3D | First order | Intratumor | 0.43 |
| Kurtosis | 3D | First order | Intratumor | 0.37 |
| **Maximum** | 3D | First order | Intratumor | 0.83 |
| Mean absolute deviation | 3D | First order | Intratumor | 0.45 |
| Mean | 3D | First order | Intratumor | 0.89 |
| Median | 3D | First order | Intratumor | 0.93 |
| Minimum | 3D | First order | Intratumor | 0.43 |
| Range | 3D | First order | Intratumor | 0.48 |
| Robust mean absolute deviation | 3D | First order | Intratumor | 0.41 |
| Root mean squared | 3D | First order | Intratumor | 0.82 |
| Skewness | 3D | First order | Intratumor | 0.71 |
| Total energy | 3D | First order | Intratumor | 0.73 |
| Uniformity | 3D | First order | Intratumor | 0.73 |
| Variance | 3D | First order | Intratumor | 0.39 |
| Autocorrelation | 3D | GLCM | Intratumor | 0.26 |
| Cluster prominence | 3D | GLCM | Intratumor | 0.31 |
| Cluster shade | 3D | GLCM | Intratumor | 0.46 |
| Cluster tendency | 3D | GLCM | Intratumor | 0.41 |
| Contrast | 3D | GLCM | Intratumor | 0.65 |
| Correlation | 3D | GLCM | Intratumor | 0.68 |
| Difference average | 3D | GLCM | Intratumor | 0.74 |
| Difference entropy | 3D | GLCM | Intratumor | 0.72 |
| Difference variance | 3D | GLCM | Intratumor | 0.50 |
| Inverse difference | 3D | GLCM | Intratumor | 0.86 |
| Inverse difference moment | 3D | GLCM | Intratumor | 0.88 |
| Inverse difference moment normalized | 3D | GLCM | Intratumor | 0.45 |
| Inverse difference | 3D | GLCM | Intratumor | 0.48 |
| Informational measure of correlation 1 | 3D | GLCM | Intratumor | 0.71 |
| Informational measure of correlation 2 | 3D | GLCM | Intratumor | 0.72 |
| Inverse variance | 3D | GLCM | Intratumor | 0.87 |
| Joint average | 3D | GLCM | Intratumor | 0.24 |
| Joint energy | 3D | GLCM | Intratumor | 0.81 |
| Joint entropy | 3D | GLCM | Intratumor | 0.74 |
| **Maximal correlation coefficient** | 3D | GLCM | Intratumor | 0.56 |
| Maximum probability | 3D | GLCM | Intratumor | 0.87 |
| Sum average | 3D | GLCM | Intratumor | 0.24 |
| Sum entropy | 3D | GLCM | Intratumor | 0.70 |
| Sum of squares | 3D | GLCM | Intratumor | 0.41 |
| Dependence entropy | 3D | GLDM | Intratumor | 0.70 |
| Dependence non-uniformity | 3D | GLDM | Intratumor | 0.95 |
| Dependence non-uniformity normalized | 3D | GLDM | Intratumor | 0.89 |
| Dependence variance | 3D | GLDM | Intratumor | 0.89 |
| Gray level non-uniformity | 3D | GLDM | Intratumor | 0.98 |
| Gray level variance | 3D | GLDM | Intratumor | 0.39 |
| High gray level emphasis | 3D | GLDM | Intratumor | 0.28 |
| Large dependence emphasis | 3D | GLDM | Intratumor | 0.91 |
| Large dependence high gray level emphasis | 3D | GLDM | Intratumor | 0.22 |
| Large dependence low gray level emphasis | 3D | GLDM | Intratumor | 0.42 |
| Low gray level emphasis | 3D | GLDM | Intratumor | 0.09 |
| Small dependence emphasis | 3D | GLDM | Intratumor | 0.86 |
| Small dependence high gray level emphasis | 3D | GLDM | Intratumor | 0.49 |
| Small dependence low gray level emphasis | 3D | GLDM | Intratumor | 0.00 |
| Gray level non-uniformity | 3D | GLRLM | Intratumor | 0.98 |
| Gray level non-uniformity normalized | 3D | GLRLM | Intratumor | 0.72 |
| Gray level variance | 3D | GLRLM | Intratumor | 0.39 |
| High gray level run emphasis | 3D | GLRLM | Intratumor | 0.28 |
| Long run emphasis | 3D | GLRLM | Intratumor | 0.90 |
| Long run high gray level emphasis | 3D | GLRLM | Intratumor | 0.23 |
| Long run low gray level emphasis | 3D | GLRLM | Intratumor | 0.19 |
| Low gray level run emphasis | 3D | GLRLM | Intratumor | 0.05 |
| Run entropy | 3D | GLRLM | Intratumor | 0.64 |
| Run length non-uniformity | 3D | GLRLM | Intratumor | 0.93 |
| Run length non-uniformity normalized | 3D | GLRLM | Intratumor | 0.89 |
| Run percentage | 3D | GLRLM | Intratumor | 0.89 |
| Run variance | 3D | GLRLM | Intratumor | 0.90 |
| Short run emphasis | 3D | GLRLM | Intratumor | 0.89 |
| Short run high gray level emphasis | 3D | GLRLM | Intratumor | 0.29 |
| Short run low gray level emphasis | 3D | GLRLM | Intratumor | 0.02 |
| **Gray level non-uniformity** | 3D | GLSZM | Intratumor | 0.98 |
| Gray level non-uniformity normalized | 3D | GLSZM | Intratumor | 0.49 |
| Gray level variance | 3D | GLSZM | Intratumor | 0.31 |
| High gray level zone emphasis | 3D | GLSZM | Intratumor | 0.32 |
| Large area emphasis | 3D | GLSZM | Intratumor | 0.94 |
| Large area high gray level emphasis | 3D | GLSZM | Intratumor | 0.59 |
| Large area low gray level emphasis | 3D | GLSZM | Intratumor | 0.14 |
| Low gray level zone emphasis | 3D | GLSZM | Intratumor | 0.00 |
| Size zone non-uniformity | 3D | GLSZM | Intratumor | 0.94 |
| Size zone non-uniformity normalized | 3D | GLSZM | Intratumor | 0.90 |
| **Small area emphasis** | 3D | GLSZM | Intratumor | 0.90 |
| Small area high gray level emphasis | 3D | GLSZM | Intratumor | 0.35 |
| Small area low gray level emphasis | 3D | GLSZM | Intratumor | 0.00 |
| Zone entropy | 3D | GLSZM | Intratumor | 0.70 |
| Zone percentage | 3D | GLSZM | Intratumor | 0.87 |
| **Zone variance** | 3D | GLSZM | Intratumor | 0.94 |
| Busyness | 3D | NGTDM | Intratumor | 0.67 |
| **Coarseness** | 3D | NGTDM | Intratumor | 0.87 |
| Complexity | 3D | NGTDM | Intratumor | 0.72 |
| Contrast | 3D | NGTDM | Intratumor | 0.42 |
| Strength | 3D | NGTDM | Intratumor | 0.63 |
| **10th percentile** | 2D | First order | Peritumor | 0.71 |
| 90th percentile | 2D | First order | Peritumor | 0.61 |
| Energy | 2D | First order | Peritumor | 0.63 |
| Entropy | 2D | First order | Peritumor | 0.64 |
| Interquartile range | 2D | First order | Peritumor | 0.56 |
| Kurtosis | 2D | First order | Peritumor | 0.49 |
| **Maximum** | 2D | First order | Peritumor | 0.78 |
| Mean absolute deviation | 2D | First order | Peritumor | 0.68 |
| Mean | 2D | First order | Peritumor | 0.51 |
| Median | 2D | First order | Peritumor | 0.49 |
| **Minimum** | 2D | First order | Peritumor | 0.87 |
| Range | 2D | First order | Peritumor | 0.77 |
| Robust mean absolute deviation | 2D | First order | Peritumor | 0.63 |
| Root mean squared | 2D | First order | Peritumor | 0.53 |
| Skewness | 2D | First order | Peritumor | 0.58 |
| Total energy | 2D | First order | Peritumor | 0.63 |
| Uniformity | 2D | First order | Peritumor | 0.65 |
| Variance | 2D | First order | Peritumor | 0.74 |
| Autocorrelation | 2D | GLCM | Peritumor | 0.39 |
| Cluster prominence | 2D | GLCM | Peritumor | 0.77 |
| **Cluster shade** | 2D | GLCM | Peritumor | 0.73 |
| Cluster tendency | 2D | GLCM | Peritumor | 0.70 |
| Contrast | 2D | GLCM | Peritumor | 0.88 |
| Correlation | 2D | GLCM | Peritumor | 0.48 |
| Difference average | 2D | GLCM | Peritumor | 0.88 |
| Difference entropy | 2D | GLCM | Peritumor | 0.87 |
| **Difference variance** | 2D | GLCM | Peritumor | 0.86 |
| Inverse difference | 2D | GLCM | Peritumor | 0.87 |
| Inverse difference moment | 2D | GLCM | Peritumor | 0.87 |
| **Inverse difference moment normalized** | 2D | GLCM | Peritumor | 0.67 |
| Inverse difference normalized | 2D | GLCM | Peritumor | 0.71 |
| Informational measure of correlation 1 | 2D | GLCM | Peritumor | 0.45 |
| Informational measure of correlation 2 | 2D | GLCM | Peritumor | 0.39 |
| Inverse variance | 2D | GLCM | Peritumor | 0.90 |
| Joint average | 2D | GLCM | Peritumor | 0.51 |
| Joint energy | 2D | GLCM | Peritumor | 0.74 |
| Joint entropy | 2D | GLCM | Peritumor | 0.73 |
| **Maximal correlation coefficient** | 2D | GLCM | Peritumor | 0.51 |
| Maximum probability | 2D | GLCM | Peritumor | 0.88 |
| Sum average | 2D | GLCM | Peritumor | 0.51 |
| Sum entropy | 2D | GLCM | Peritumor | 0.64 |
| Sum of squares | 2D | GLCM | Peritumor | 0.73 |
| Dependence entropy | 2D | GLDM | Peritumor | 0.56 |
| Dependence non-uniformity | 2D | GLDM | Peritumor | 0.84 |
| Dependence non-uniformity normalized | 2D | GLDM | Peritumor | 0.85 |
| Dependence variance | 2D | GLDM | Peritumor | 0.83 |
| Gray level non-uniformity | 2D | GLDM | Peritumor | 0.51 |
| Gray level variance | 2D | GLDM | Peritumor | 0.73 |
| High gray level emphasis | 2D | GLDM | Peritumor | 0.43 |
| Large dependence emphasis | 2D | GLDM | Peritumor | 0.81 |
| Large dependence high gray level emphasis | 2D | GLDM | Peritumor | 0.24 |
| Large dependence low gray level emphasis | 2D | GLDM | Peritumor | 0.94 |
| Low gray level emphasis | 2D | GLDM | Peritumor | 0.95 |
| Small dependence emphasis | 2D | GLDM | Peritumor | 0.84 |
| Small dependence high gray level emphasis | 2D | GLDM | Peritumor | 0.53 |
| Small dependence low gray level emphasis | 2D | GLDM | Peritumor | 0.89 |
| Gray level non-uniformity | 2D | GLRLM | Peritumor | 0.52 |
| Gray level non-uniformity normalized | 2D | GLRLM | Peritumor | 0.65 |
| Gray level variance | 2D | GLRLM | Peritumor | 0.73 |
| High gray level run emphasis | 2D | GLRLM | Peritumor | 0.45 |
| Long run emphasis | 2D | GLRLM | Peritumor | 0.82 |
| Long run high gray level emphasis | 2D | GLRLM | Peritumor | 0.39 |
| Long run low gray level emphasis | 2D | GLRLM | Peritumor | 0.95 |
| Low gray level run emphasis | 2D | GLRLM | Peritumor | 0.94 |
| Run entropy | 2D | GLRLM | Peritumor | 0.62 |
| Run length non-uniformity | 2D | GLRLM | Peritumor | 0.82 |
| Run length non-uniformity normalized | 2D | GLRLM | Peritumor | 0.82 |
| Run percentage | 2D | GLRLM | Peritumor | 0.82 |
| Run variance | 2D | GLRLM | Peritumor | 0.81 |
| Short run emphasis | 2D | GLRLM | Peritumor | 0.82 |
| Short run high gray level emphasis | 2D | GLRLM | Peritumor | 0.46 |
| Short run low gray level emphasis | 2D | GLRLM | Peritumor | 0.93 |
| Gray level non-uniformity | 2D | GLSZM | Peritumor | 0.51 |
| Gray level non-uniformity normalized | 2D | GLSZM | Peritumor | 0.62 |
| Gray level variance | 2D | GLSZM | Peritumor | 0.73 |
| High gray level zone emphasis | 2D | GLSZM | Peritumor | 0.47 |
| Large area emphasis | 2D | GLSZM | Peritumor | 0.81 |
| Large area high gray level emphasis | 2D | GLSZM | Peritumor | 0.27 |
| **Large area low gray level emphasis** | 2D | GLSZM | Peritumor | 0.93 |
| Low gray level zone emphasis | 2D | GLSZM | Peritumor | 0.90 |
| Size zone non-uniformity | 2D | GLSZM | Peritumor | 0.86 |
| Size zone non-uniformity normalized | 2D | GLSZM | Peritumor | 0.84 |
| Small area emphasis | 2D | GLSZM | Peritumor | 0.83 |
| Small area high gray level emphasis | 2D | GLSZM | Peritumor | 0.51 |
| Small area low gray level emphasis | 2D | GLSZM | Peritumor | 0.85 |
| Zone entropy | 2D | GLSZM | Peritumor | 0.53 |
| Zone percentage | 2D | GLSZM | Peritumor | 0.84 |
| Zone variance | 2D | GLSZM | Peritumor | 0.82 |
| Busyness | 2D | NGTDM | Peritumor | 0.59 |
| **Coarseness** | 2D | NGTDM | Peritumor | 0.62 |
| Complexity | 2D | NGTDM | Peritumor | 0.91 |
| Contrast | 2D | NGTDM | Peritumor | 0.78 |
| **Strength** | 2D | NGTDM | Peritumor | 0.82 |
| 10th percentile | 3D | First order | Peritumor | 0.94 |
| 90th percentile | 3D | First order | Peritumor | 0.75 |
| **Energy** | 3D | First order | Peritumor | 0.78 |
| Entropy | 3D | First order | Peritumor | 0.78 |
| Interquartile range | 3D | First order | Peritumor | 0.82 |
| Kurtosis | 3D | First order | Peritumor | 0.76 |
| **Maximum** | 3D | First order | Peritumor | 0.83 |
| Mean absolute deviation | 3D | First order | Peritumor | 0.81 |
| Mean | 3D | First order | Peritumor | 0.77 |
| Median | 3D | First order | Peritumor | 0.83 |
| Minimum | 3D | First order | Peritumor | 0.98 |
| Range | 3D | First order | Peritumor | 0.81 |
| Robust mean absolute deviation | 3D | First order | Peritumor | 0.80 |
| Root mean squared | 3D | First order | Peritumor | 0.77 |
| Skewness | 3D | First order | Peritumor | 0.79 |
| Total energy | 3D | First order | Peritumor | 0.77 |
| Uniformity | 3D | First order | Peritumor | 0.78 |
| Variance | 3D | First order | Peritumor | 0.84 |
| Autocorrelation | 3D | GLCM | Peritumor | 0.84 |
| Cluster prominence | 3D | GLCM | Peritumor | 0.88 |
| **Cluster shade** | 3D | GLCM | Peritumor | 0.87 |
| Cluster tendency | 3D | GLCM | Peritumor | 0.82 |
| Contrast | 3D | GLCM | Peritumor | 0.90 |
| Correlation | 3D | GLCM | Peritumor | 0.83 |
| Difference average | 3D | GLCM | Peritumor | 0.88 |
| Difference entropy | 3D | GLCM | Peritumor | 0.86 |
| Difference variance | 3D | GLCM | Peritumor | 0.91 |
| Inverse difference | 3D | GLCM | Peritumor | 0.87 |
| Inverse difference moment | 3D | GLCM | Peritumor | 0.87 |
| **Inverse difference moment normalized** | 3D | GLCM | Peritumor | 0.72 |
| Inverse difference normalized | 3D | GLCM | Peritumor | 0.75 |
| Informational measure of correlation 1 | 3D | GLCM | Peritumor | 0.73 |
| Informational measure of correlation 2 | 3D | GLCM | Peritumor | 0.73 |
| Inverse variance | 3D | GLCM | Peritumor | 0.90 |
| Joint average | 3D | GLCM | Peritumor | 0.87 |
| Joint energy | 3D | GLCM | Peritumor | 0.80 |
| Joint entropy | 3D | GLCM | Peritumor | 0.81 |
| **Maximal correlation coefficient** | 3D | GLCM | Peritumor | 0.81 |
| Maximum probability | 3D | GLCM | Peritumor | 0.90 |
| Sum average | 3D | GLCM | Peritumor | 0.87 |
| Sum entropy | 3D | GLCM | Peritumor | 0.79 |
| Sum of squares | 3D | GLCM | Peritumor | 0.84 |
| Dependence entropy | 3D | GLDM | Peritumor | 0.77 |
| Dependence non-uniformity | 3D | GLDM | Peritumor | 0.94 |
| Dependence non-uniformity normalized | 3D | GLDM | Peritumor | 0.89 |
| Dependence variance | 3D | GLDM | Peritumor | 0.91 |
| Gray level non-uniformity | 3D | GLDM | Peritumor | 0.71 |
| Gray level variance | 3D | GLDM | Peritumor | 0.84 |
| High gray level emphasis | 3D | GLDM | Peritumor | 0.83 |
| Large dependence emphasis | 3D | GLDM | Peritumor | 0.86 |
| **Large dependence high gray level emphasis** | 3D | GLDM | Peritumor | 0.90 |
| Large dependence low gray level emphasis | 3D | GLDM | Peritumor | 0.94 |
| Low gray level emphasis | 3D | GLDM | Peritumor | 0.96 |
| Small dependence emphasis | 3D | GLDM | Peritumor | 0.88 |
| Small dependence high gray level emphasis | 3D | GLDM | Peritumor | 0.82 |
| Small dependence low gray level emphasis | 3D | GLDM | Peritumor | 0.96 |
| Gray level non-uniformity | 3D | GLRLM | Peritumor | 0.72 |
| Gray level non-uniformity normalized | 3D | GLRLM | Peritumor | 0.79 |
| Gray level variance | 3D | GLRLM | Peritumor | 0.84 |
| High gray level run emphasis | 3D | GLRLM | Peritumor | 0.83 |
| Long run emphasis | 3D | GLRLM | Peritumor | 0.85 |
| Long run high gray level emphasis | 3D | GLRLM | Peritumor | 0.83 |
| Long run low gray level emphasis | 3D | GLRLM | Peritumor | 0.95 |
| Low gray level run emphasis | 3D | GLRLM | Peritumor | 0.96 |
| Run entropy | 3D | GLRLM | Peritumor | 0.78 |
| Run length non-uniformity | 3D | GLRLM | Peritumor | 0.89 |
| Run length non-uniformity normalized | 3D | GLRLM | Peritumor | 0.86 |
| Run percentage | 3D | GLRLM | Peritumor | 0.85 |
| Run variance | 3D | GLRLM | Peritumor | 0.85 |
| Short run emphasis | 3D | GLRLM | Peritumor | 0.86 |
| Short run high gray level emphasis | 3D | GLRLM | Peritumor | 0.83 |
| Short run low gray level emphasis | 3D | GLRLM | Peritumor | 0.96 |
| Gray level non-uniformity | 3D | GLSZM | Peritumor | 0.81 |
| Gray level non-uniformity normalized | 3D | GLSZM | Peritumor | 0.71 |
| Gray level variance | 3D | GLSZM | Peritumor | 0.84 |
| High gray level zone emphasis | 3D | GLSZM | Peritumor | 0.82 |
| Large area emphasis | 3D | GLSZM | Peritumor | 0.41 |
| **Large area high gray level emphasis** | 3D | GLSZM | Peritumor | 0.60 |
| Large area low gray level emphasis | 3D | GLSZM | Peritumor | 0.17 |
| Low gray level zone emphasis | 3D | GLSZM | Peritumor | 0.91 |
| Size zone non-uniformity | 3D | GLSZM | Peritumor | 0.96 |
| Size zone non-uniformity normalized | 3D | GLSZM | Peritumor | 0.90 |
| Small area emphasis | 3D | GLSZM | Peritumor | 0.89 |
| Small area high gray level emphasis | 3D | GLSZM | Peritumor | 0.82 |
| Small area low gray level emphasis | 3D | GLSZM | Peritumor | 0.91 |
| Zone entropy | 3D | GLSZM | Peritumor | 0.70 |
| Zone percentage | 3D | GLSZM | Peritumor | 0.87 |
| Zone variance | 3D | GLSZM | Peritumor | 0.41 |
| Busyness | 3D | NGTDM | Peritumor | 0.75 |
| **Coarseness** | 3D | NGTDM | Peritumor | 0.80 |
| Complexity | 3D | NGTDM | Peritumor | 0.98 |
| Contrast | 3D | NGTDM | Peritumor | 0.81 |
| Strength | 3D | NGTDM | Peritumor | 0.95 |
