# Supplementary material for: Role of intratumoral and peritumoral CT radiomics for the prediction of EGFR gene mutation in primary lung cancer
Source: Br J Radiol. 2022 Sep 22;95(1140):20220374. doi: 10.1259/bjr.20220374 (PMC9733609; doi:10.1259/bjr.20220374)
Supplement: Supplementary Table 1. [file bjr.20220374.suppl-02.docx]

***Table S2: Feature importance***

| **Rank** | **Feature name** | **Dimension** | **Feature family** | **Extracted region** | **Importance *** |
| --- | --- | --- | --- | --- | --- |
| 1 | Brinkman index | N. A. | Clinical | N. A. | 19.57 |
| 2 | 90th percentile | 3D | First order | Intratumor | 13.59 |
| 3 | Sex | N. A. | Clinical | N. A. | 11.49 |
| 4 | Large area high gray level emphasis | 3D | GLSZM | Peritumor | 10.25 |
| 5 | Maximal correlation coefficient | 3D | GLCM | Intratumor | 6.53 |
| 6 | Inverse difference moment normalized | 2D | GLCM | Peritumor | 6.46 |
| 7 | Cluster shade | 2D | GLCM | Intratumor | 6.21 |
| 8 | Inverse difference moment normalized | 3D | GLCM | Peritumor | 5.52 |
| 9 | Difference variance | 2D | GLCM | Peritumor | 5.47 |
| 10 | Coarseness | 3D | NGTDM | Intratumor | 4.91 |
| 11 | Cluster shade | 3D | GLCM | Peritumor | 4.87 |
| 12 | Maximum | 2D | First order | Intratumor | 4.83 |
| 13 | Large dependence high gray level emphasis | 3D | GLDM | Peritumor | 4.52 |
| 14 | Gray level non-uniformity | 3D | GLSZM | Intratumor | 4.49 |
| 15 | Zone variance | 3D | GLSZM | Intratumor | 4.26 |
| 16 | Energy | 3D | First order | Intratumor | 4.22 |
| 17 | Maximum | 3D | First order | Peritumor | 4.12 |
| 18 | Elongation | 2D | Shape | Intratumor | 4.05 |
| 19 | Sphericity | 3D | Shape | Intratumor | 4.01 |
| 20 | Small area emphasis | 3D | GLSZM | Intratumor | 3.96 |
| 21 | Maximum | 3D | First order | Intratumor | 3.89 |
| 22 | Maximal correlation coefficient | 3D | GLCM | Peritumor | 3.85 |
| 23 | Coarseness | 2D | NGTDM | Peritumor | 3.83 |
| 24 | Strength | 2D | NGTDM | Peritumor | 3.76 |
| 25 | Large area low gray level emphasis | 2D | GLSZM | Intratumor | 3.73 |
| 26 | Age | N. A. | Clinical | N. A. | 3.58 |
| 27 | Maximum | 2D | First order | Peritumor | 3.52 |
| 28 | Elongation | 3D | Shape | Intratumor | 3.40 |
| 29 | Large area low gray level emphasis | 2D | GLSZM | Peritumor | 3.34 |
| 30 | Coarseness | 3D | NGTDM | Peritumor | 3.28 |
| 31 | Energy | 3D | First order | Peritumor | 3.22 |
| 32 | 10th percentile | 2D | First order | Peritumor | 3.21 |
| 33 | Maximal correlation coefficient | 2D | GLCM | Peritumor | 3.15 |
| 34 | Cluster shade | 2D | GLCM | Peritumor | 2.62 |
| 35 | Minimum | 2D | First order | Peritumor | 1.80 |
| 36 | Clinical stage | N. A. | N. A. | Clinical | 0.70 |

* Measured using the mean decrease in the Gini index.

GLCM, gray level co-occurrence matrix; GLDM, gray level dependence matrix; GLSZM, gray level size zone matrix; NGTDM, neighboring gray tone difference matrix
