# Supplementary material for: Role of intratumoral and peritumoral CT radiomics for the prediction of EGFR gene mutation in primary lung cancer
Source: Br J Radiol. 2022 Sep 22;95(1140):20220374. doi: 10.1259/bjr.20220374 (PMC9733609; doi:10.1259/bjr.20220374)
Supplement: Supplementary Table 2. [file bjr.20220374.suppl-03.docx]

***Overview of decision tree and Random Forest***

Figure S1 below illustrates an example of a decision tree. Decision trees split input datasets according to the optimal cutoff point of a given feature and provide the probability of an event of interest (EGFR mutation rate in this study). Once a tree is constructed using training data, it can make relevant predictions based on new data that were not used in its construction. Output probability is determined by where the data is classified (red, blue, green, and yellow parts in Figure S1).

Figure S1: Example of decision tree


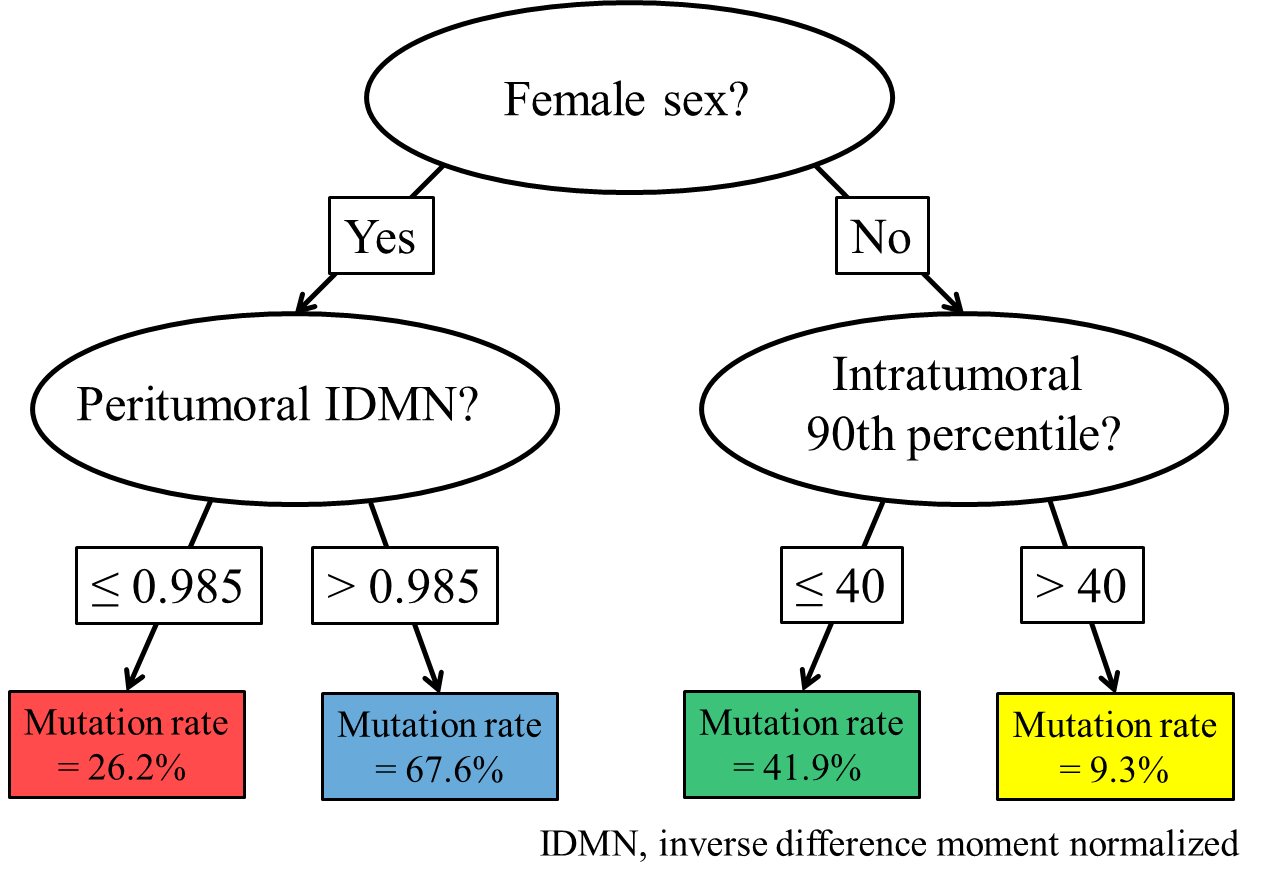


Although a single decision tree is a good classifier, the accuracy can be improved by combining predictions from a greater number of decision trees. Random Forest (Figure S2, next page) is an algorithm that combines many decision trees (e.g., 500 trees). Furthermore, Random Forest can calculate the feature importance.

In Random Forest, training data used to construct each decision tree is generated by random resampling from the entire dataset. The number of times random sampling is performed is the same as the number of the entire dataset. Therefore, the number of training data becomes the same as that of the entire dataset. Because of the resampling, the same data may be selected more than once into training data. Statistically, about one-third of the entire dataset is not selected as training data. The unselected data are called out-of-bag (OOB) data and are used to test the performance of each tree. Training data and OOB data are created for each decision tree. Accordingly, each decision tree uses different training and OOB data.

Figure S3 (next page) shows an example of training data and OOB data when the number of the entire dataset is six. Since the number of datasets in this study is 478, the number of training data for each decision tree is 478, and that of OOB data is 478/3 ≈ 159. A detail on decision trees and Random Forest can be found in the following two papers:

1. Kingsford C, Salzberg SL. What are decision trees? Nat Biotechnol. 2008; 26: 1011–3
2. Breiman L. Random Forests. Machine Learning 2001; 45: 5–32

Figure S2: Random Forest consisted of 500 decision trees

***
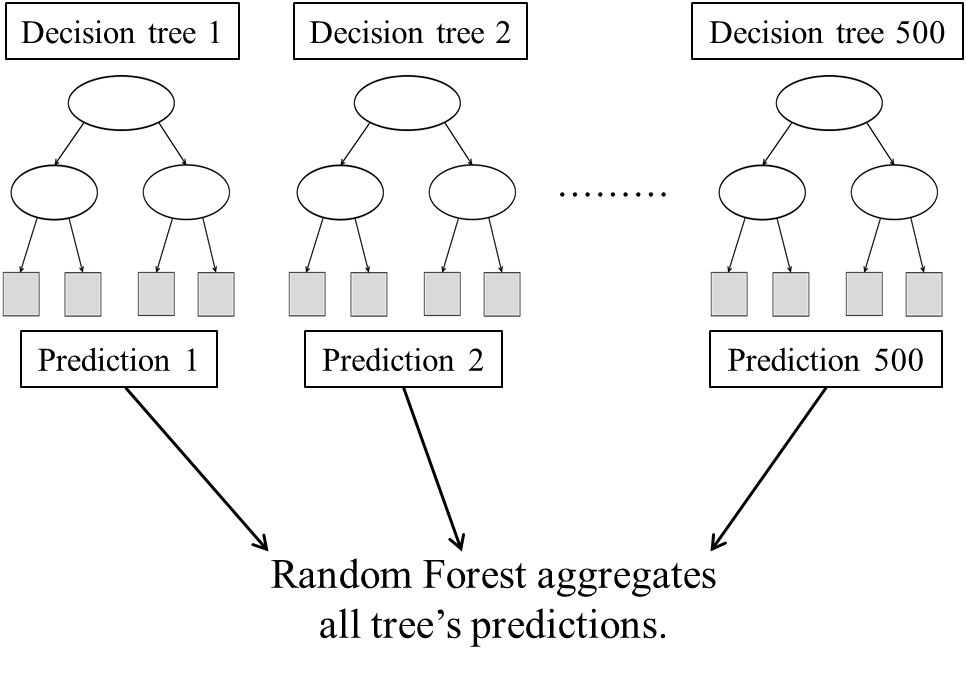
***

Figure S3: An example of training data and OOB data divided using sampling with replacement

***
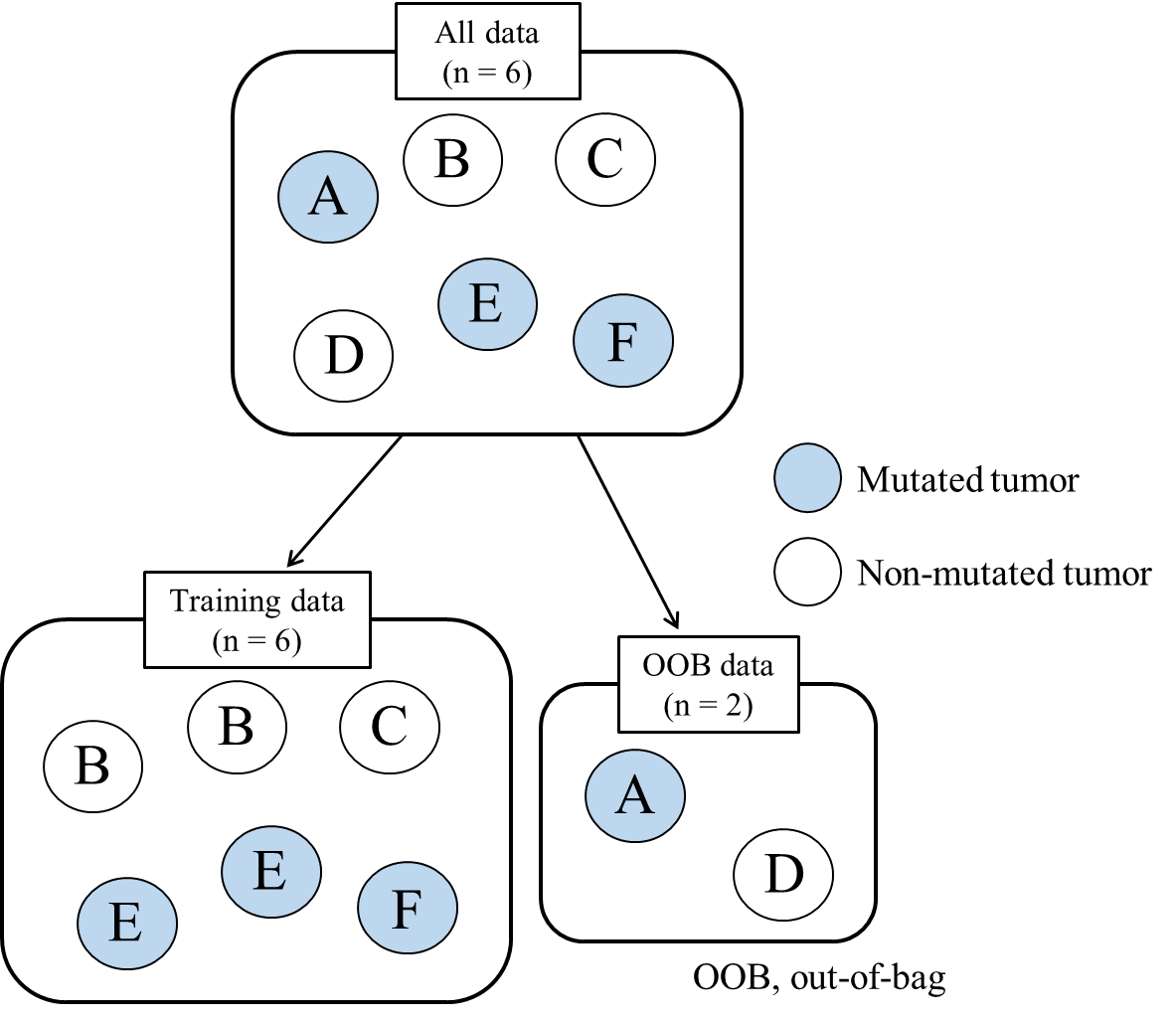
***

***Segmentation workflow***

This section provides a detailed procedure for tumor and peritumoral segmentation using 3D Slicer. Lung segmentation was also performed to prevent leakage of the peritumoral segmentation into the extrapulmonary structures, such as the chest wall and mediastinum. The segmentation was performed in a semi-automatic manner using　"**Segmentation Editor**" module and "**SegmentEditorExtraEffects**" effect (Figure S4). If necessary, segmentation was manually modified.

1. Image cropping
   Before segmentation, high-resolution CT images were cropped to a small size that contains only the necessary region using "**Crop Volume**" module (Figure S5 and S6). Cropping images reduces the computation time for subsequent operations.
2. Semi-automatic tumor segmentation
   The tumor was manually segmented on every two to three axial slices (Figure S7); skipped slices were automatically interpolated using the operation, "**Fill between slices**" (Figure S8).
3. Semi-automatic lung segmentation
   1. Air in the lung, defined as CT values ≤ -500 Hounsfield units (HU), was automatically segmented by "**Local threshold**" effect (Figure 9S). This effect was performed using the following settings.
      - Threshold range: -1024 HU ≤, ≤ -500 HU
      - Minimum diameter: 5 mm
      - Segmentation algorithm: GrowCut
      - Editable area: Outside all segments
      - Modify other segments: Allow overlap
   2. Subsequently, pulmonary vessels and bronchi were included into the above segmentation by "**Smoothing**" effect (Figure 10S), with the settings below:
      - Smoothing method: Closing (fill holes)
      - Kernel size: 10 mm
      - Editable area: Outside all segments
      - Modify other segments: Allow overlap
4. Semi-automatic segmentation of the peritumoral region

The tumor segment (green segment) was copied to another empty segment by using "**Logical operators**" effect, and the copied segment was automatically converted to a lung segment within a distance of 3 mm from the tumor border by using "**Hollow**" effect. This segment (yellow segment) was defined as peritumoral region in the present study (Figure 11S). The operation for "**Hollow**" effect was set as below:

- Use current segment as: inside surface
- Shell thickness: 3 mm
- Editable area: Inside all segments
- Modify other segments: Allow overlap

Figure S4: Segmentation toolbar in 3D Slicer


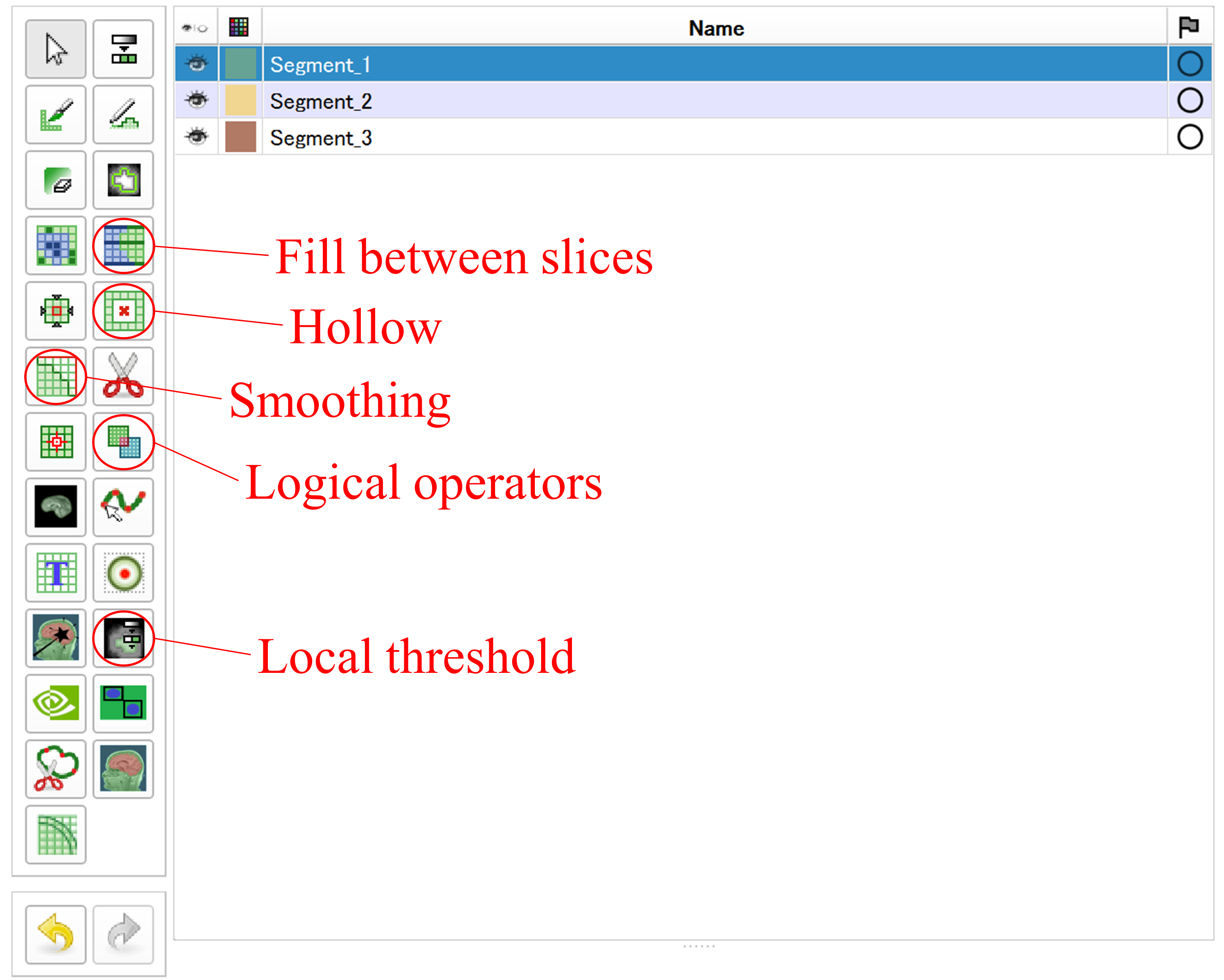


Figure S5: Original image

Axial view Coronal view


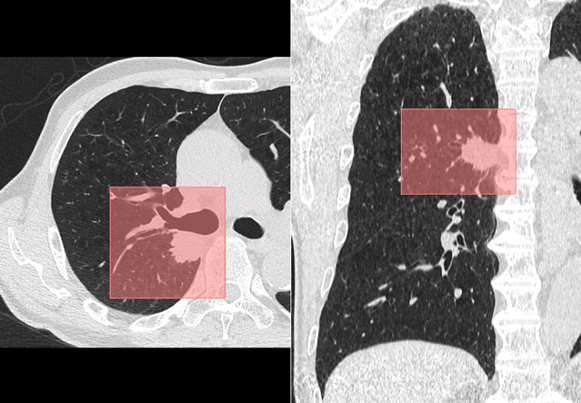


Figure S6: Cropped image

Axial view Coronal view


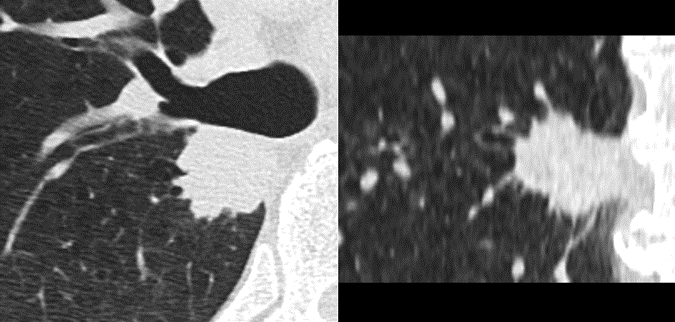


Figure S7: Before interpolation

Axial view Coronal view


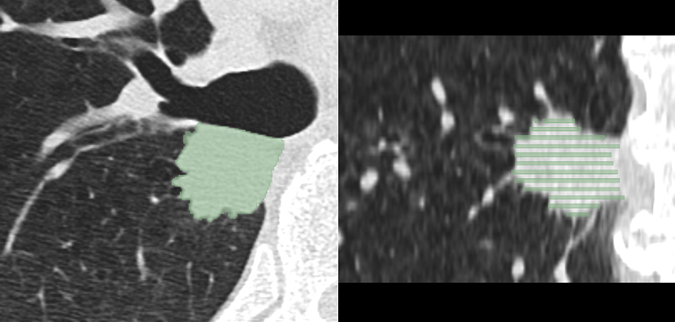


Figure S8: After interpolation

Axial view Coronal view


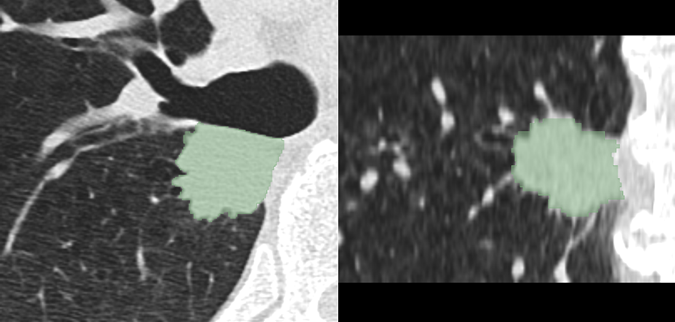


Figure S9: Lung segmentation before including vessels and bronchi

Axial view Coronal view


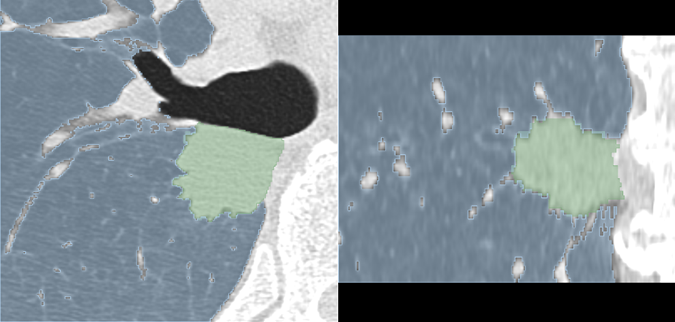


Figure S10: Lung segmentation after including vessels and bronchi

Axial view Coronal view


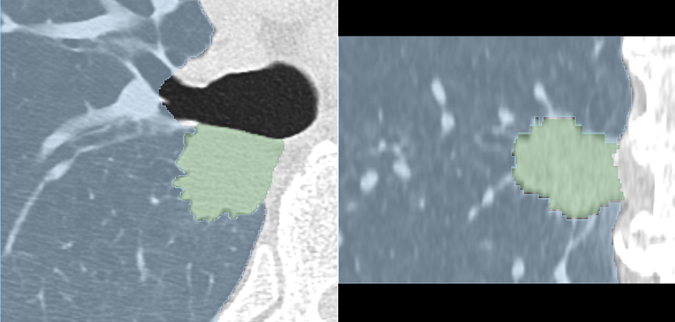


Figure S11: Segmentation of peritumoral region

Axial view Coronal view


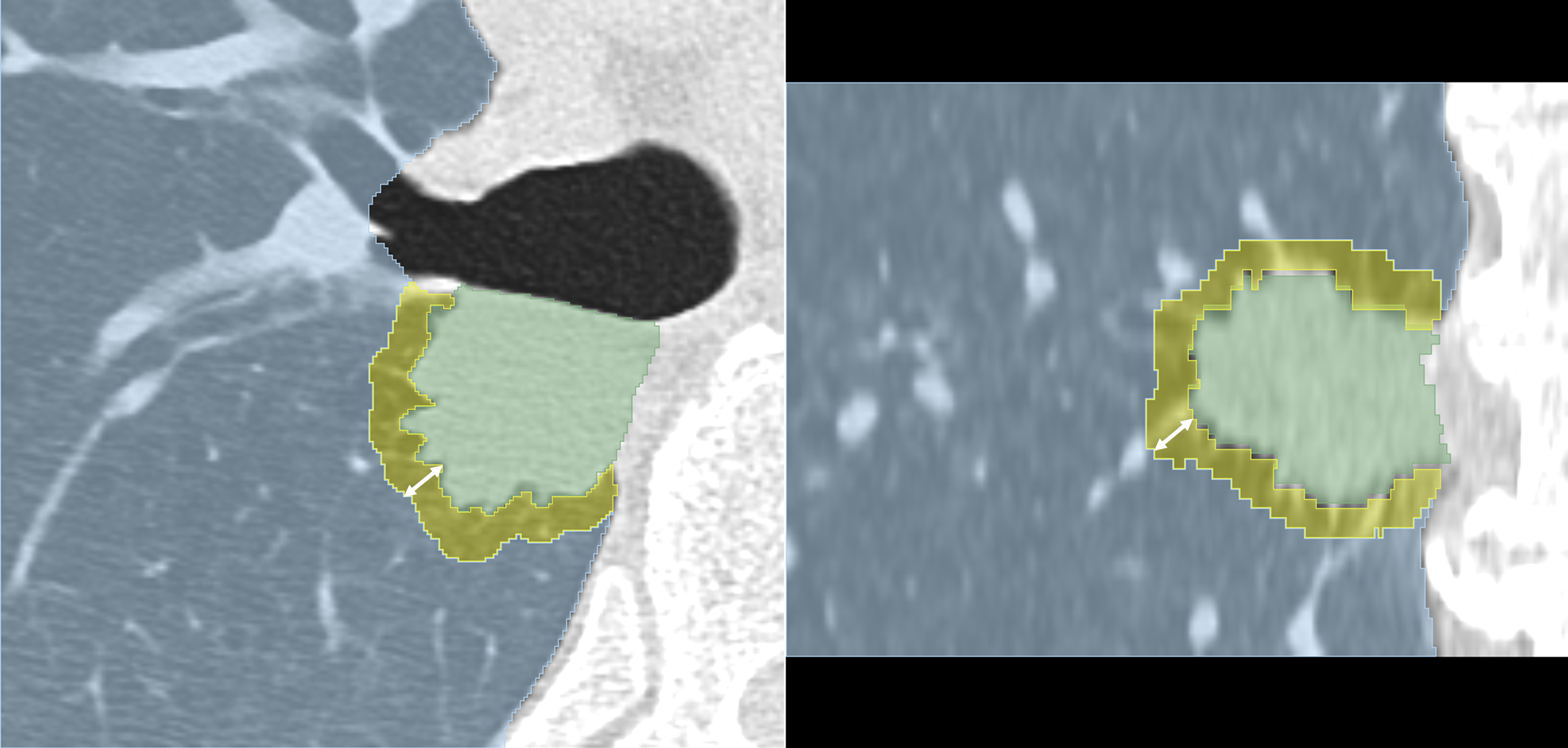


3mm

3mm
